# Heparan sulfate is essential for thymus growth

**DOI:** 10.1016/j.jbc.2021.100419

**Published:** 2021-02-15

**Authors:** Hsuan-Po Hsu, Yun-Tzu Chen, Yu-Ying Chen, Chih-Yu Lin, Po-Yu Chen, Shio-Yi Liao, Ciara Christianne Y. Lim, Yu Yamaguchi, Chia-Lin Hsu, Ivan L. Dzhagalov

**Affiliations:** 1 Institute of Microbiology and Immunology, National Yang-Ming University, Taipei, Taiwan; 2 Sanford Burnham Prebys Medical Discovery Institute, La Jolla, California, USA

**Keywords:** heparan sulfate, fibroblasts, thymus, chemokines, T cell development, DC, dendritic cell, DAPI, 4′,6-diamidino-2-phenylindole, DN, CD4^-^CD8^-^ double-negative, DP, CD4^+^CD8^+^ double-positive, EC, endothelial cell, ECM, extracellular matrix, FTOC, fetal thymic organ culture, Glce, Glucoronyl C5-Epimerase, HS, heparan sulfate, HSase, Heparinase, K5, Keratin 5, mBMDC, mature bone-marrow-derived dendritic cell, mTEC, medullary thymic epithelial cell, PBS, phosphate buffered saline, SP, CD4^+^CD8^-^ or CD4^-^CD8^+^ single-positive, TEC, thymic epithelial cell, TN, CD4^-^CD8^-^TCR^-^ triple-negative

## Abstract

Thymus organogenesis and T cell development are coordinated by various soluble and cell-bound molecules. Heparan sulfate (HS) proteoglycans can interact with and immobilize many soluble mediators, creating fields or gradients of secreted ligands. While the role of HS in the development of many organs has been studied extensively, little is known about its function in the thymus. Here, we examined the distribution of HS in the thymus and the effect of its absence on thymus organogenesis and T cell development. We found that HS was expressed most abundantly on the thymic fibroblasts and at lower levels on endothelial, epithelial, and hematopoietic cells. To study the function of HS in the thymus, we eliminated most of HS in this organ by genetically disrupting the glycosyltransferase Ext1 that is essential for its synthesis. The absence of HS greatly reduced the size of the thymus in fetal thymic organ cultures and *in vivo*, in mice, and decreased the production of T cells. However, no specific blocks in T cell development were observed. Wild-type thymic fibroblasts were able to physically bind the homeostatic chemokines CCL19, CCL21, and CXCL12 *ex vivo*. However, this binding was abolished upon HS degradation, disrupting the CCL19/CCL21 chemokine gradients and causing impaired migration of dendritic cells in thymic slices. Thus, our results show that HS plays an essential role in the development and growth of the thymus and in regulating interstitial cell migration.

T cell development in the thymus occurs through a series of well-choreographed steps that result in the generation of major histocompatibility complex-restricted and self-tolerant repertoire of cells that can protect us from pathogen invasion. Successful transition of thymocytes between developmental stages requires input by various supporting cell types such as dendritic cells (DCs), macrophages, thymic epithelial cells (TECs), and fibroblasts. For example, the commitment of the most immature progenitors to the T cell lineage requires Notch ligands expressed on epithelial cells (1, 2); positive selection requires self-peptide–major histocompatibility complex complexes expressed on cortical TECs; efficient negative selection needs promiscuous gene expression by medullary TECs (mTECs) and antigen presentation by DCs (3, 4). In addition to these cell-associated signals, thymocytes also need many soluble factors produced, mostly, by nonhematopoietic cells that maintain their viability, such as IL-7 (5), or instruct their migration to microenvironments supporting their differentiation such as the chemokines CCL19, CCL21, CCL25, and CXCL12 (6). The bone-marrow-derived precursors are attracted to the thymus by these four chemokines (7, 8, 9) and enter the organ at the junction between the outer part, the cortex, and the inner part, the medulla (10). These most immature progenitors do not express the coreceptors CD4 and CD8 and are called double-negative (DN) thymocytes. They are guided to the subcapsular zone of the cortex by CCL25 (11). After successfully rearranging their TCRβ chains, these cells upregulate CD4 and CD8 becoming double-positive (DP) cells. DP thymocytes are the most abundant cell population in the thymus, and they occupy the majority of the cortex. They are thought to be retained there by the action of CXCL12 (12, 13). Cells that successfully rearrange the TCRα chain and pass positive selection then migrate to the medulla under the influence of CCR7 ligands, CCL19 and CCL21 (14), and perhaps CCR4 ligands, CCL17 and CCL22 (15). In the process, they downregulate CD4 or CD8 and become lineage-committed CD4^+^CD8^-^ or CD4^-^CD8^+^ single-positive (SP) cells. The importance of chemokines for successful T cell development is best illustrated in mice lacking CCL19 and CCL21 (plt mice) or CCR7-deficient mice, which do not efficiently migrate to the medulla following positive selection and fail to undergo negative selection to tissue-restricted antigens resulting in an autoimmune disease (16).

Fibroblasts are an important, yet relatively poorly understood, component of the thymus. They are cells of mesenchymal origin derived from the neural crest (17, 18) and are the primary producers of the extracellular matrix (ECM) in the organ. Fibroblasts, together with epithelial cells, are the first components of the thymus anlage and play important roles in thymus organogenesis. In addition to providing structural foundation by building the capsule and the septae of the organ, they also ensheath the blood vessels (19) and elaborate growth factors, such as Fgf7, Fgf10, Igf1, and Igf2 and retinoic acid for epithelial cells (20, 21). Removal of the fibroblasts at embryonic day E12 from the thymus anlage leads to defective expansion of TECs and growth retardation of the whole organ (22). Yet, even under these conditions, the pattern of T cell development was not changed, suggesting that fibroblasts regulate it only indirectly through affecting epithelial cells proliferation. However, recently, fibroblasts were pointed out as an important source of self-antigen for the induction of central tolerance, suggesting that they can directly influence T cell development (23). Unfortunately, few studies have examined the effects on thymocytes of gene disruption, specifically in thymic fibroblasts (23, 24, 25, 26). Thus, the role of fibroblast in the thymus is still unclear.

Heparan sulfate (HS) is a negatively charged, linear, polysaccharide composed of alternating N-acetylated or N-sulfated glucosamine units and uronic (glucuronic or iduronic) acid. It is a major component of the ECM in all organs, and it is also present on the surface of many cells attached to proteins such as Syndecans and Glypicans (27). The main enzymes responsible for the HS polymer synthesis are the glycosyltransferases Ext1 and Ext2 (28). In the absence of Ext1, no HS is produced, and mice lacking Ext1 die during gastrulation (E6.5–8.5) before organs form, underscoring the importance of HS in a variety of developmental processes (29). The biological importance of HS lies in its ability to interact with more than 300 secreted proteins, including cytokines, chemokines, growth factors, and morphogens. Binding of these ligands modifies their functions through immobilization, protection from degradation, and facilitation of their oligomerization and interaction with other proteins (30).

Within the immune system, the role of HS has been studied in several different contexts. Many chemokines and cytokines that play important roles for the homeostasis of the immune system or during an immune response, such as CCL19, CCL21, CXCL12, IL-7, IL-2, IFNβ, CXCL2, and CXCL8, have been shown to interact with HS (31, 32, 33, 34, 35, 36, 37). Genetic ablation of some of the enzymes involved in the synthesis or modification of HS has demonstrated that it plays critical roles in the extravasation of leukocytes (31, 37, 38) and the trafficking of DCs to lymph nodes (39, 40). Moreover, deficiency of Glucuronyl C5-Epimerase (Glce), an enzyme that controls the flexibility of HS chains, leads to defective development of the lymphoid organs (41) and impaired final stages of B cell maturation (42). HS on macrophages regulates IFNβ signaling and protects against obesity and atherosclerosis (35). Thus, HS regulates many aspects of immune system development and function. However, the role of HS in thymus organogenesis and T cell development has received only limited attention. The only indication that HS might play a role in formation of the thymus comes from studies of Glce^-/-^ mice that revealed defects in thymus positioning and lobulation without impaired T cell development (41). Unfortunately, Glce^-/-^ mice die at birth, and these studies could not be extended to adult mice. HS interacts with many of the chemokines and cytokines that are important for T cell development in the thymus, suggesting that it can play a vital role in the process. Intrinsic deficiency of HS modifying enzymes Ndst1 and Ndst2 in T cells does not lead to gross changes in T cell homeostasis (43). However, two recent studies showed that mutations in Extl3, another enzyme participating in the synthesis of HS, lead to T cell deficiency, suggesting that HS regulates T cell development in the thymus (44, 45).

In this study, we examined the importance of HS for thymus organogenesis and T cell development. The finding that HS expression is primarily restricted to thymic fibroblasts allowed us to devise a genetic strategy to eliminate the majority of HS in the organ. We explored the effects of HS absence using fetal thymic organ cultures (FTOCs) and embryonic thymus transplantation. Both methods showed a significant reduction in the size of the thymus, but a relatively normal developmental pattern of thymocytes. Part of the growth retardation could be attributed to defective interaction of HS with homeostatic chemokines that led to impaired interstitial migration of immune cells within the thymus. Thus, our study demonstrates that HS regulates thymus organogenesis, at least in part through facilitating motility inside the organ.

## Results

### HS is produced predominantly by fibroblasts in the thymus

**Figure 1:**
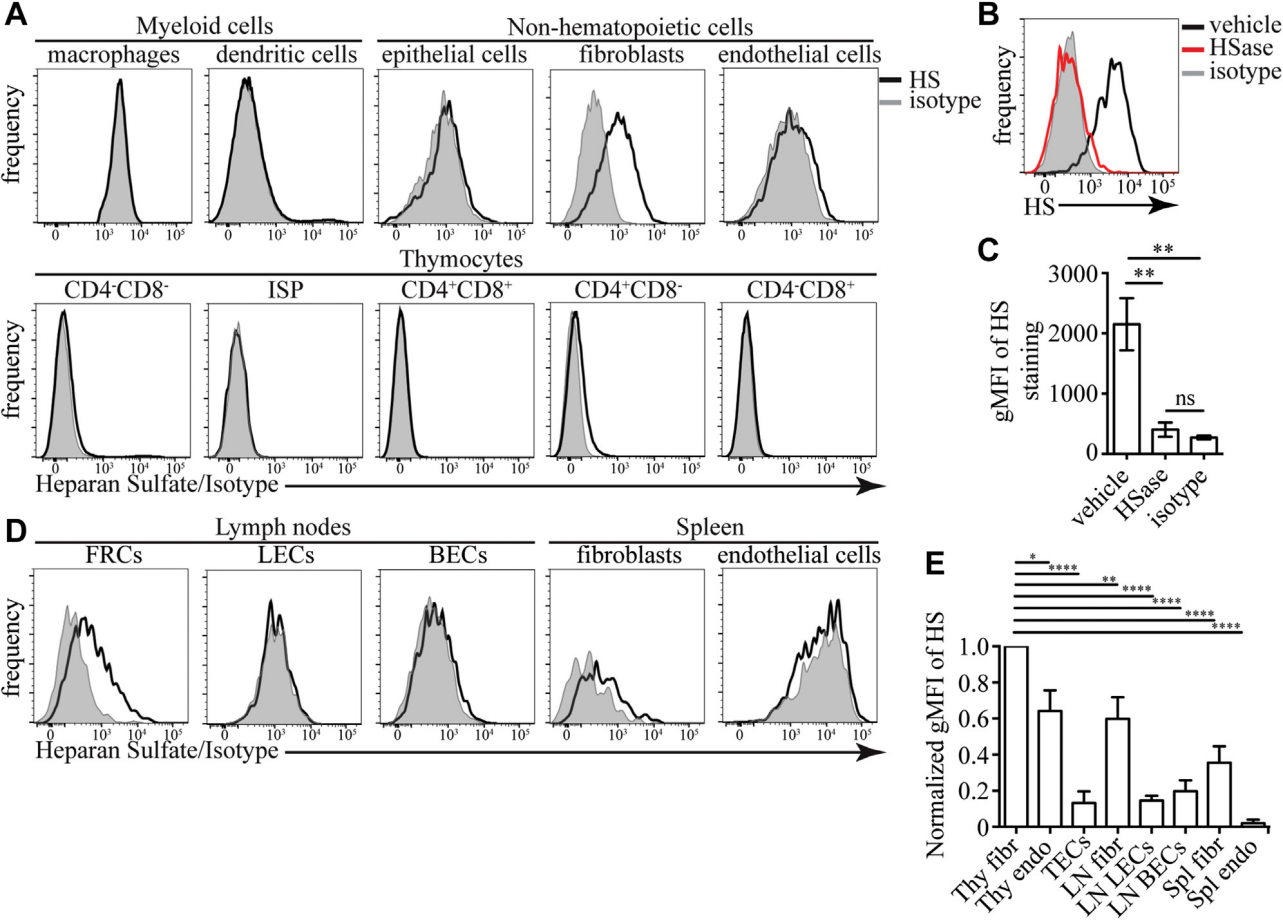
**Expression of HS in the thymus and other lymphoid organs detected with 10E4 antibody.***A*, flow cytometric detection of HS in various cell types in the thymus (F4/80^+^ macrophages, CD11c^+^MHC2^+^ dendritic cells, CD45^-^EpCAM^+^ epithelial cells, CD45^-^gp38^+^ fibroblasts, CD45^-^CD31^+^ ECs, DN, DP, CD4SP, CD8SP, and CD8^+^CD4^-^TCR immature SP (ISP) thymocytes). The results are representative of more than three independent experiments. *B*, example flow cytometry plots for HS staining on CD45^-^EpCAM^-^gp38^+^ thymic fibroblasts after treatment with Heparinase (HSase) or vehicle. For comparison, the staining of isotype control antibody is shown (*gray*). *C*, quantification of the geometric mean fluorescent intensity (gMFI) of HS staining on thymic fibroblasts after treatment with HSase or vehicle. The results are mean ± SEM from six independent experiments. *D*, expression of HS on nonhematopoietic cells in lymph nodes CD45^-^CD31^-^gp38^+^ fibroblastic reticular cells (FRCs), CD45^-^CD31^+^gp38^+^ lymphatic endothelial cells (LECs), and CD45^-^CD31^+^gp38^-^ blood endothelial cells (BECs) and spleen CD45^-^PDGFRα^+^ fibroblasts and CD45^-^CD31^+^ ECs. The results are representative of three independent experiments. *E*, comparison of the amount of HS on the surfaces of the indicated nonhematopoietic cells. For each experiment, the gMFI for each cell type was normalized to the gMFI of thymic fibroblasts that was set to 1. The results are mean ± SEM from 3 to 5 independent experiments. Statistical significance in C and *E*, was determined with unpaired *t*-test. ∗*p* < 0.05, ∗∗*p* < 0.01, ∗∗∗∗*p* < 0.0001, ns, not significant. MHC, major histocompatibility complex.

We decided to characterize HS expression on the various cells within the thymus as a first step to understand its role in this organ. We used the 10E4 antibody, which recognizes a common HS epitope (46), to stain thymus single-cell suspension and examined the binding by flow cytometry (see Fig. S1 for gating strategy). HS was most abundant on CD45^-^EpCAM^-^gp38^+^ thymic fibroblasts (Fig. 1*A*). CD45^-^EpCAM^+^ TECs, CD31^+^ endothelial cells (ECs), and CD4SP thymocytes had lower HS on their surface. We could not detect HS on F4/80^+^ macrophages, thymic DCs, DN, immature single-positive cells (ISPs), DP, and CD8SP thymocytes. Confirming the antibody’s specificity, digestion of HS with Heparinase (HSase) completely abolished the staining (Fig. 1, *B* and *C*). As the 10E4 antibody does not recognize all possible HS structures, we confirmed our findings with the 3G10 antibody that recognizes all HS chains after digestion with HSase. Staining with 3G10 revealed that all cells in the thymus express HS (Fig. S2, *A* and *B*). However, the hierarchy of the expression levels was the same as uncovered with the 10E4 antibody; namely, fibroblasts had the highest HS expression, followed by ECs, and the hematopoietic cells. The fibroblasts’ ability to produce HS was not limited to the thymus, as lymph node (LN) gp38^+^CD31^-^ fibroblasts and spleen PDGFRα^+^ fibroblasts were also positive for this glycosaminoglycan (Fig. 1*D*). When we quantified HS expression levels on different cell types, we found that thymic fibroblasts expressed significantly higher amounts of HS than any other cell type that we have examined (Fig. 1*E*).

To confirm that the glycosyltransferases *Ext1* and *Ext2*, which are responsible for the extension of the HS chain, are highly expressed in fibroblasts, we mined the IMMGEN database (www.immgen.org). In agreement with our flow cytometry data, the catalytic unit, *Ext1*, was expressed at the highest level in fibroblasts and, in particular, thymic fibroblasts (Fig. S3*A*). Among thymocytes, CD4SP expressed the highest levels of *Ext1*. The expression in other thymocyte populations and mTECs was relatively low. The chaperone unit, *Ext2*, did not vary as much among the different cell types but had the highest expression level in fibroblasts. We also used the RNA-Seq data from a recently published paper that examined different thymus fibroblast populations together with TECs, thymic ECs, and LN fibroblasts (23). This analysis confirmed that *Ext1* and *Ext2* are most highly expressed in thymus fibroblasts, followed by LN fibroblasts, ECs, and TECs (Fig. S3*B*). Both capsular and medullary fibroblasts expressed comparable levels of *Ext1* and *Ext2* mRNA. Based on RNA-Seq data and flow cytometry, we conclude that fibroblasts are the primary producers of HS in the thymus and other lymphoid organs.

**Figure 2:**
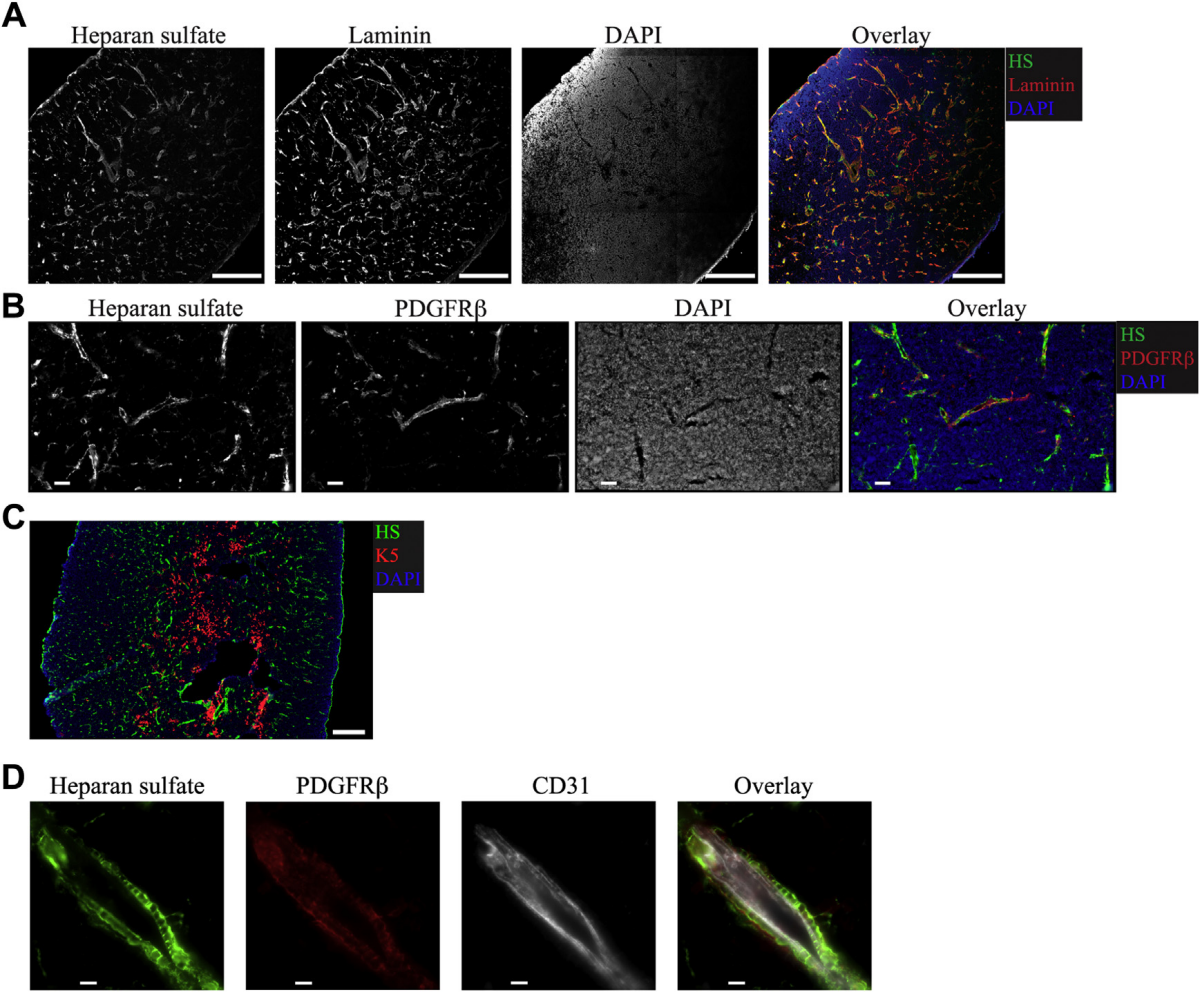
**HS localization around blood vessels and in the capsule of the thymus.***A*, immunofluorescent (IF) staining for HS and Laminin on thymic tissue sections. DAPI is used to counterstain the nuclei. Scale bar is 200 μm. *B*, IF staining showing colocalization of HS with PDGFRβ, a marker for fibroblasts, in thymic sections. Scale bar is 50 μm. *C*, IF staining of thymic tissue sections for the mTEC marker Keratin 5 (K5) and HS. Scale bar is 200 μm. *D*, IF staining of thymic tissue sections for the fibroblast marker PDGFRβ, endothelial cell marker CD31, and HS. Scale bar is 10 μm. The images are representative of at least three independent experiments. DAPI, 4′,6-diamidino-2-phenylindole

Immunofluorescent staining for HS revealed a perivascular and capsular staining pattern that colocalized with Laminin (Fig. 2*A*), a protein enriched in the basement membrane of blood vessels (47). This result suggests that HS is enriched around blood vessels, where most fibroblasts are located (19). To confirm this, we stained thymic sections with antibodies to HS and PDGFRβ, a marker for fibroblasts (48). Indeed, HS and PDGFRβ signal colocalized to a great extent (Fig. 2*B*). In contrast, there was minimal overlap between Keratin 5 (K5), a marker for medullary thymic epithelial cells, and HS (Fig. 2*C*). Colocalization between HS and ECs was more challenging to measure because of the close apposition between ECs and the fibroblasts that ensheath them. Still, higher magnification images showed that HS staining overlapped better with PDGFRβ^+^ fibroblasts than with CD31^+^ ECs (Fig. 2*D*). Thus, our data show that HS is produced mostly by fibroblasts in the thymus and is localized predominantly around the blood vessels and in the organ’s capsule.

### HS ablation in Pdgfrα^Cre^ X Ext1^f/f^ mice leads to multiple developmental abnormalities and embryonic death

**Figure 3:**
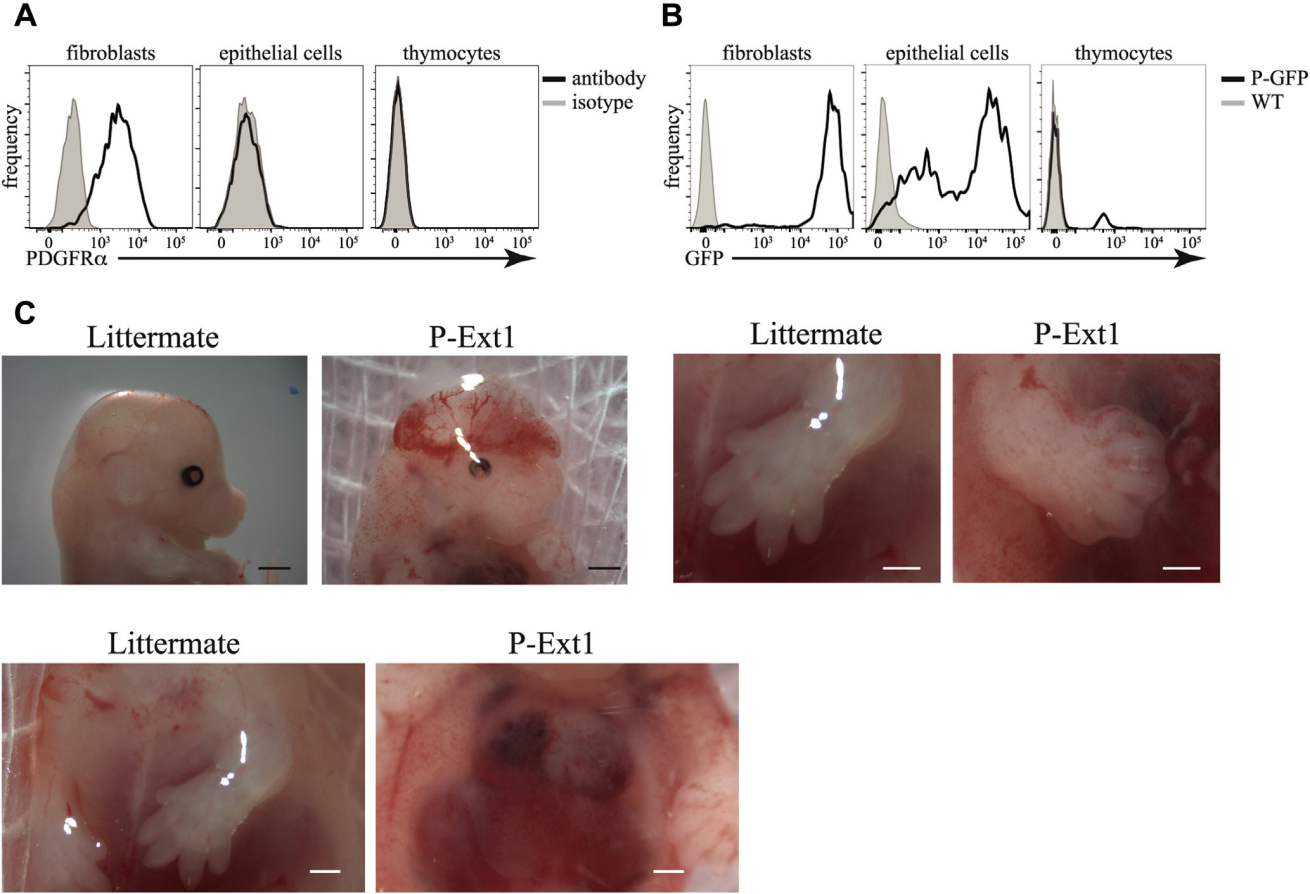
**Deletion of Ext1 in Pdgfrα**^**Cre**^**expressing cells.***A*, expression of PDGFRα in fibroblasts, epithelial cells, and thymocytes determined by flow cytometry. Isotype antibody staining serves as a background control. *B*, GFP expression in the same cell types in Pdgfrα^Cre^ X R26-LSL-GFP (P-GFP) mice. C57BL/6 mice (WT) serve as a background control. *C*, examples of some of the abnormalities in Pdgfrα^Cre^ X Ext1 (P-Ext1) embryos, such as exencephaly (*top*, *left*, *scale bars* are 1 mm), failure of the digits to separate (*top*, *right*, *scale bars* are 0.5 mm), and failure of the rib cage to close (*bottom*, *scale bars* are 0.5 mm). Littermates missing Pdgfrα^Cre^ serve as controls. The results in *A* and *B*, are representative of at least three independent experiments. The results in *C* are representative of more than 20 embryos.

To study the importance of HS for T cell development and thymus organogenesis, we wanted to develop a genetic model of HS absence in the thymus. Mice lacking HS die very early during embryogenesis (29), underscoring the importance of this glycosaminoglycan for development. Thus, we decided to take a conditional knockout approach. Because of the highest abundance of HS on fibroblasts in the thymus, we reasoned that if we ablate its production in fibroblasts, the amount of HS in the organ will be greatly reduced, generating, in essence, an HS-deficient thymus. As PDGFRα is expressed on almost all fibroblasts in the thymus (48) but not on TECs or thymocytes (Fig. 3*A*), we decided to use Pdgfrα^Cre^ mice (49) to achieve conditional deletion in fibroblasts. To verify the fidelity of Pdgfrα^Cre^, we crossed these mice to the ROSA26-LSL-GFP line (50). In their progeny, expression of Cre is marked by GFP fluorescence. As we expected, virtually all thymic fibroblasts expressed GFP (Fig. 3*B*), indicating that Pdgfrα^Cre^ is an excellent tool to delete genes in fibroblasts. Surprisingly, a large proportion of TECs and a small fraction of thymocytes were also GFP-positive, indicating an unfaithful expression of Cre or that ECs go through a PDGFRα expressing stage. Regardless, the absence of HS on additional cell types in the thymus could only aid our goal of producing mice with HS-deficient thymus.

To ablate HS on the thymic fibroblasts and other cells in which Pdgfrα^Cre^ is active, we crossed Pdgfrα^Cre^ mice to Ext1^f/f^ mice (51). Given the importance of HS for embryonic development and the presence of PDGFRα^+^ fibroblasts in many organs, it was not surprising to find that Pdgfrα^Cre^ X Ext1^f/f^ (P-Ext1) mice died *in utero*. We screened 68 embryos between E12.5 and E19.5 and found only six of the P-Ext1 genotype. The oldest viable conditional knockout embryos were recovered at E14.5. The mutant embryos presented with numerous abnormalities such as exencephaly, webbed digits, failure of the rib cage to close (Fig. 3*C*), demonstrating that the absence of HS in cells expressing Pdgfrα^Cre^ is essential for many developmental processes.

### Normal T cell development in FTOCs lacking HS

**Figure 4:**
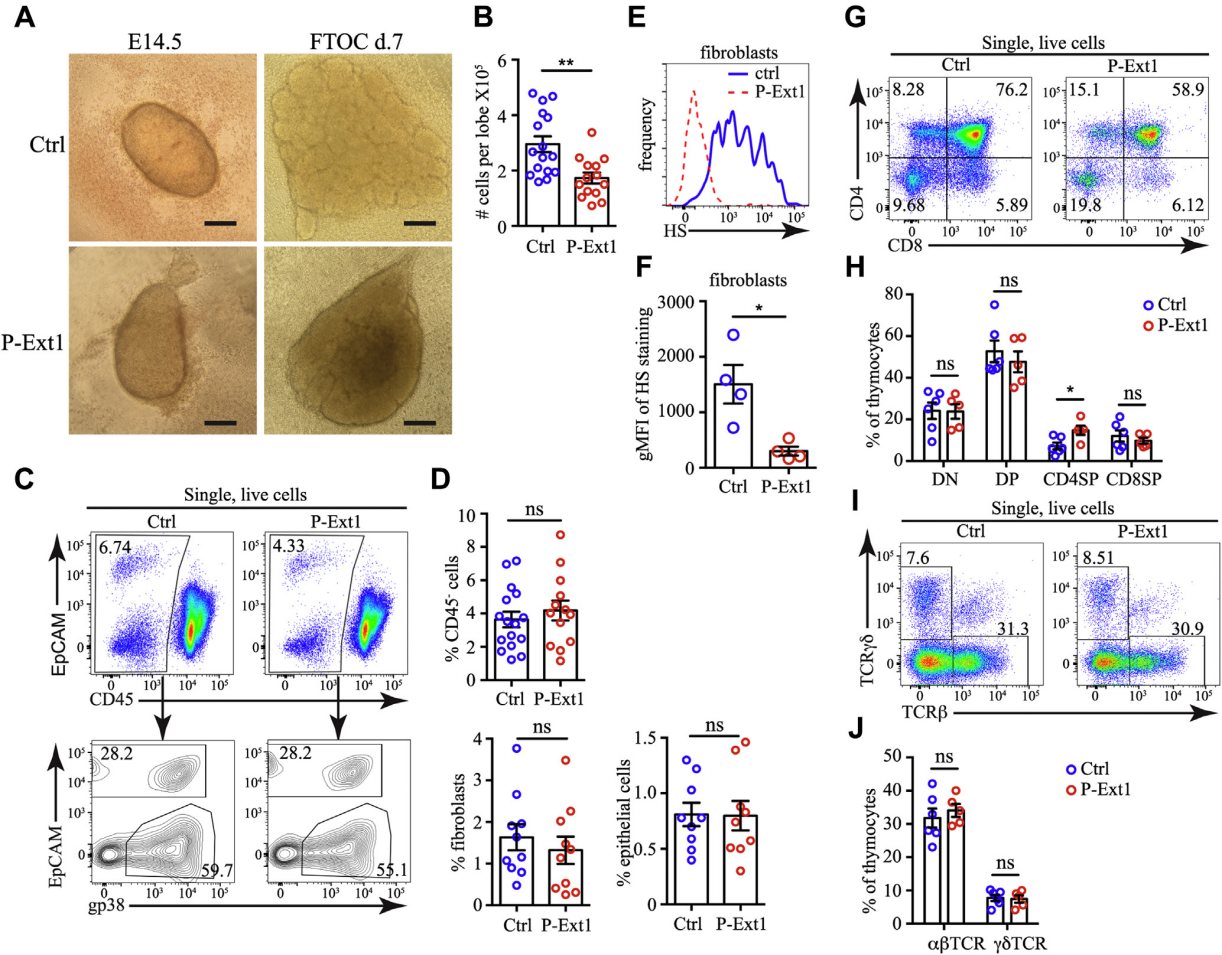
**Smaller size, but no block in T cells development in P-Ext1 FTOCs.***A*, photographs of E14.5 embryonic thymuses and day 7 FTOC cultures from control and P-Ext1 embryos. The images are representative of 16 ctrl and 14 P-Ext1 embryonic thymuses at their isolation and after 7 days of culture. *Scale bar* is 0.2 mm. *B*, comparison of the cell numbers in P-Ext1 and control FTOCs. *C*, flow cytometry plots showing the percentage of nonhematopoietic cells (CD45^-^), as well as fibroblasts and epithelial cells in control and P-Ext1 FTOCs. *D*, frequencies of CD45^-^ cells (*top*) and of fibroblasts and epithelial cells (*bottom*) among single live cells in control and P-Ext1 FTOCs. *E*, flow cytometry plot of HS expression in fibroblasts in control (*blue*, *solid*) and P-Ext1 (*red*, *dashed*) FTOCs. *F*, comparison of the gMFI of HS in fibroblasts in control and P-Ext1 FTOCs. *G*, CD4 versus CD8 flow cytometry plots of control and P-Ext1 FTOCs showing the major thymocyte populations. The number inside each gate indicates the percentage of the respective population. *H*, frequencies of DN, DP, CD4SP, and CD8SP thymocytes in control and P-Ext1 FTOCs. *I*, TCRβ versus TCRγδ flow cytometry plots of control and P-Ext1 FTOCs showing the proportion of αβT and γδT cells. The number inside each gate indicates the percentage of the respective population. *J*, frequencies of αβT and γδT cells in control and P-Ext1 FTOCs. All flow cytometry plots are representative of at least five independent experiments. Data in *B*, *D*, *F*, *H*, and *J*. are mean ± SEM. Each *dot* is an individual mouse. Statistical significance was determined with unpaired *t*-test, ∗*p* < 0.05, ∗∗*p* < 0.01, ns, not significant.

The embryonic thymus is already formed by E14.5, and we used FTOC to study the development of P-Ext1 thymus *in vitro*. We dissected E14.5 thymuses from P-Ext1 embryos and controls and cultured them for 7 days at medium–air interface. The E14.5 embryonic thymuses of controls and P-Ext1 mice were similar in size. However, the FTOCs from P-Ext1 embryos grew slower and were less lobulated after 7 days of culture (Fig. 4*A*). Moreover, P-Ext1 FTOCs contained significantly fewer cells than controls (Fig. 4*B*). When we analyzed the stromal compartment of the thymus, we found out that P-Ext1 FTOCs had proportions of EpCAM^+^ ECs and gp38^+^ fibroblasts comparable with controls (Fig. 4, *C* and *D*). As expected, the levels of HS on fibroblasts were significantly reduced (Fig. 4, *E* and *F*), indicating efficient depletion of Ext1 in thymic fibroblasts and validating our model. Then, we examined T cell development in the FTOCs. Despite the total cellularity reduction, we observed a typical pattern of T cell development and the absence of any developmental blocks. The major subsets of thymocytes—DN, DP, and CD8SP, were represented in similar proportions in P-Ext1 and control FTOCs, although CD4SP was relatively increased in P-Ext1 FTOCs (Fig. 4, *G* and *H*). The reason for the increase in CD4SP thymocytes is unclear because the percentages of the most mature TCRβ^high^ and TCRγδ^+^ thymocytes were also equivalent between P-Ext1 and control FTOCs (Fig. 4, *I* and *J*). Thus, the loss of HS impedes the growth of P-Ext1 FTOCs but does not perturb thymocyte development.

### Retarded growth of transplanted P-Ext1 thymus without a block in T cell development

**Figure 5:**
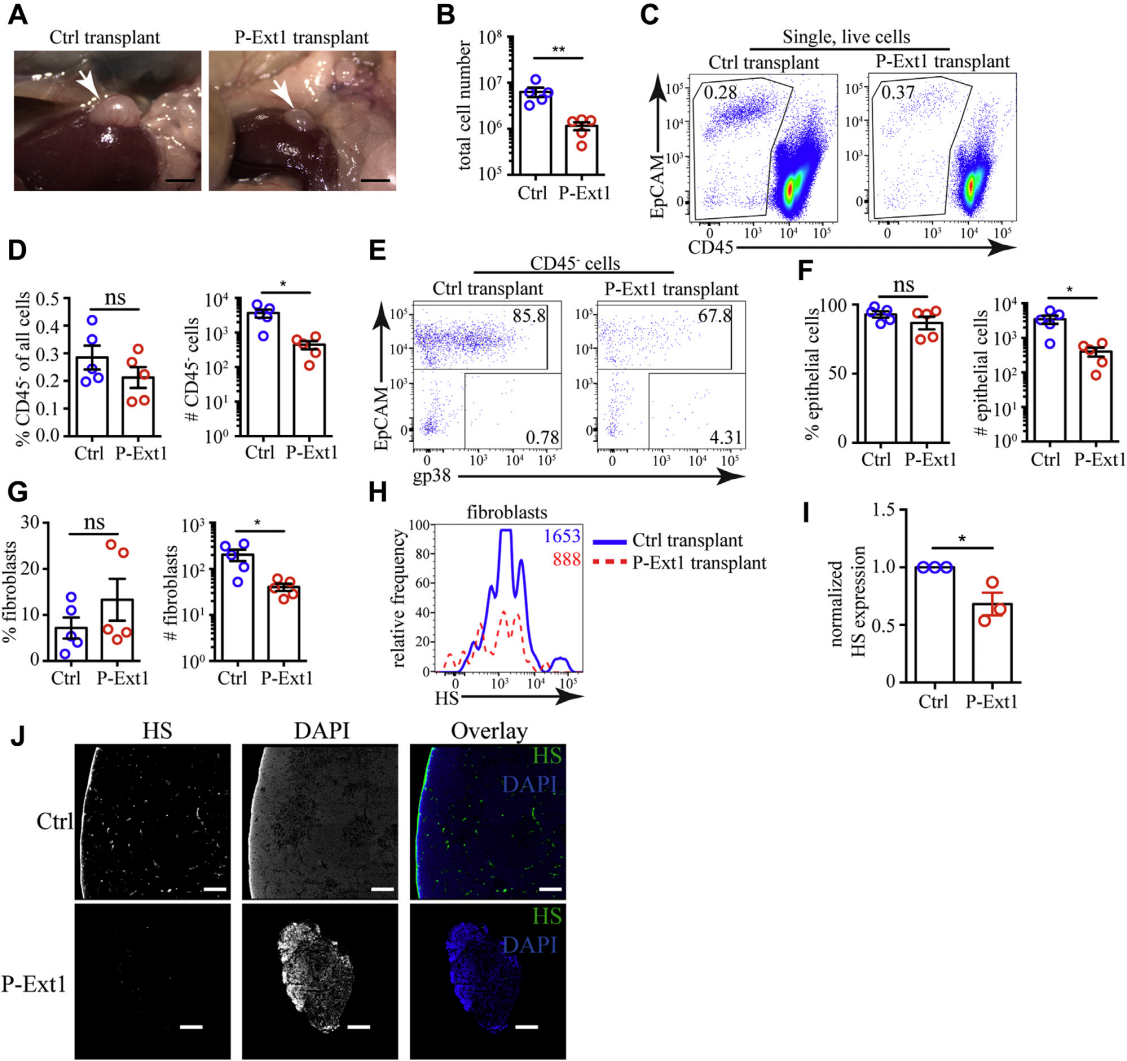
**Reduced size of P-Ext1 thymus transplant, but fibroblasts and epithelial cells are present in normal proportions.***A*, photographs of control and P-Ext1 thymuses transplanted under the kidney capsule of C57BL/6 mice 6 weeks earlier. *Scale bars* are 2 mm. *B*, comparison of the cell numbers in control and P-Ext1 transplanted thymuses. *C*, flow cytometry plots showing CD45^-^ cells in control and P-Ext1 transplanted thymuses. The numbers inside the plots are the percentages of cells in the respective gates. *D*, frequencies among single live cells and absolute numbers of CD45^-^ cells in control and P-Ext1 transplanted thymuses. *E*, flow cytometry plots showing the distribution of fibroblasts and epithelial cells among CD45^-^ cells in control and P-Ext1 transplanted thymuses. The numbers inside each plot are the percentages of cells in the respective gates. *F*, frequencies among CD45^-^ cells and absolute numbers of epithelial cells in control and P-Ext1 transplanted thymuses. *G*, frequencies among CD45^-^ cells and absolute numbers of fibroblasts in control and P-Ext1 transplanted thymuses. *H*, flow cytometry plot showing the expression of HS on fibroblasts in control (*solid*, *blue*) and P-Ext1 (*dashed*, *red*) transplanted thymuses. The numbers inside the plot are the gMFI of HS staining in fibroblasts in control (*blue*) and P-Ext1 (*red*) transplanted thymuses. *I*, comparison of the surface expression of HS revealed with 10E4 antibody on CD45^-^EpCAM^-^gp38^+^ thymic fibroblasts from control and P-Ext1 transplants. For each experiment, the gMFI of HS on fibroblasts from P-Ext1 thymus was normalized to the gMFI of HS on fibroblasts from control thymus that was set to 1. *J*, immunofluorescent staining for HS in tissue sections from control (*top row*) and P-Ext1 (*bottom row*) transplanted thymuses counterstained with DAPI. The scale bar is 150 μm. The data in *A*, *C*, and *E* are representative of five transplanted thymuses from each genotype, while the data in *H*, and *J* are representative of three different transplanted thymuses from each genotype. The data in *B*, *D*, *F*, *G*, and *I* are mean ± SEM. Each *dot* is an individual mouse. Statistical significance was determined with unpaired *t*-test, ∗*p* < 0.05, ∗∗*p* < 0.01, ns, not significant. DAPI, 4′,6-diamidino-2-phenylindole

To examine the effect of HS deficiency in the thymus in more physiological settings, we transplanted E14.5 P-Ext1 or control embryonic thymuses under the kidney capsules of C57BL/6 mice and let them grow for 6 weeks. Control transplants grew well and averaged ∼6 x 10^6^ cells per thymus; however, the P-Ext1 grafts were much smaller and contained ∼10^6^ cells (Fig. 5, *A* and *B*). Nonhematopoietic CD45^-^ cells were represented in similar proportions in P-Ext1 and control transplants (Fig. 5, *C* and *D*). The same was true for EpCAM^+^ epithelial cells (Fig. 5, *E* and *F*) and gp38^+^ fibroblasts (Fig. 5, *E* and *G*). However, the absolute numbers of all stromal cell populations were significantly reduced in P-Ext1 grafts. The abundance of HS on fibroblasts was significantly diminished in P-Ext1 transplants, as revealed by flow cytometry (Fig. 5, *H* and *I*), and HS was virtually undetectable by immunofluorescent staining of tissue sections (Fig. 5*J*). While this data does not allow us to completely rule out infiltration of the graft by host fibroblasts, even if they are present, they could not restore HS production in the transplanted thymuses to wild-type levels.

**Figure 6:**
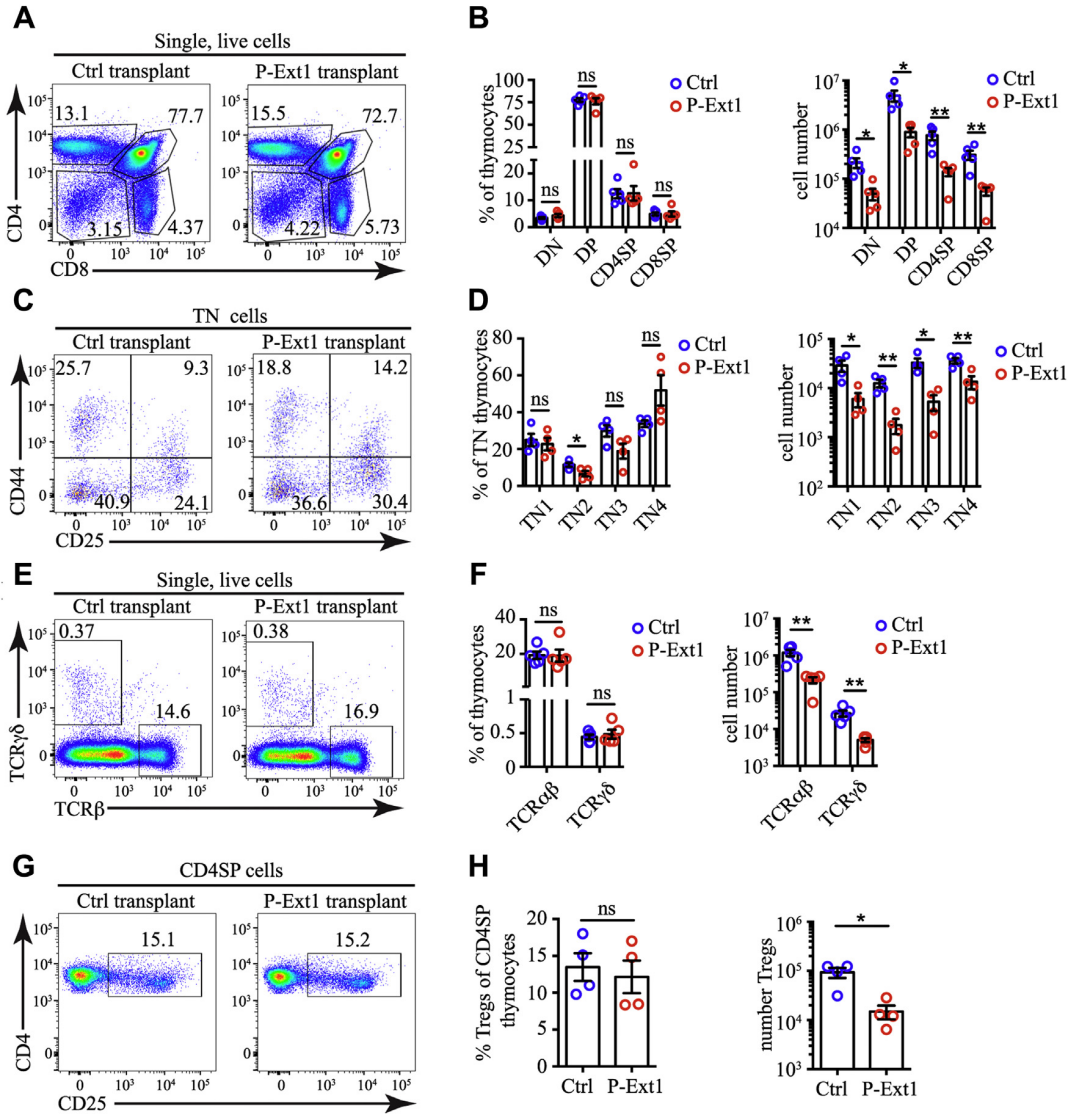
**Reduced thymocyte numbers, but no block in T cell development in P-Ext1 transplanted thymuses.***A*, CD4 versus CD8 flow cytometry plots of control and P-Ext1 transplanted thymuses showing the major thymocyte populations. *B*, frequencies among single live cells and absolute numbers of DN, DP, CD4SP, and CD8SP thymocytes in control and P-Ext1 transplanted thymuses. *C*, CD44 versus CD25 flow cytometry plots of control and P-Ext1 transplanted thymuses gated on CD4^-^CD8^-^TCR^-^ (triple negative, TN) cells. *D*, frequencies among TN cells and absolute numbers of TN1, TN2, TN3, and TN4 thymocytes among control and P-Ext1 transplanted thymuses. *E*, TCRβ versus TCRγδ flow cytometry plots of control and P-Ext1 transplanted thymuses showing the proportions of αβT and γδT cells. *F*, frequencies among single cells and absolute numbers of αβT and γδT cells in control and P-Ext1 transplanted thymuses. *G*, CD25 versus CD4 flow cytometry plots pregated on CD4SP of control and P-Ext1 transplanted thymuses showing the proportions of regulatory T cells and their precursors (Tregs). *H*, frequencies among CD4SP cells and absolute numbers of Tregs in control and P-Ext1 transplanted thymuses. The numbers inside the flow cytometry plots indicate the percentages of the cells in the respective gates. All flow cytometry plots are representative of at least four independent experiments. Data in *B*, *D*, *F*, and *H* are mean ± SEM. Each *dot* is an individual mouse. Statistical significance was determined with unpaired *t*-test, ∗*p* < 0.05, ∗∗<0.01, ns, not significant.

The T cell development pattern was preserved in P-Ext1 transplants. All major thymocyte populations, DN, DP, CD4SP, and CD8SP, were present in similar proportions compared with controls ruling out a developmental block in T cell development (Fig. 6, *A* and *B*). However, the number of cells in each subset was significantly reduced in P-Ext1 transplants. We then examined the most immature thymocytes that do not express CD4, CD8, and TCR—triple-negative (TN) cells. Based on the expression of CD25 and CD44, they can be ordered in a developmental progression starting with the most immature CD44^+^CD25^-^ TN1 cells, through CD44^+^CD25^+^ TN2, CD44^-^CD25^+^ TN3, and ending with CD44^-^CD25^-^ TN4 thymocytes. All TN subsets, except for TN2, were present in similar proportions in control and P-Ext1 grafts but were significantly reduced in numbers in P-Ext1 transplants (Fig. 6, *C* and *D*). Similarly, the most mature TCRγδ^+^ and TCRβ^hi^ cells were also equivalent in proportions but significantly reduced in numbers in P-Ext1 grafts compared with controls (Fig. 6, *E* and *F*). The same phenomenon was also apparent when we examined CD25^+^ CD4SP regulatory T cells (Fig. 6, *G* and *H*). In all cases, the proportions in P-Ext1 transplants were comparable with controls, but the absolute numbers were significantly reduced. Thus, the absence of HS in the thymus results in a smaller organ size but a typical pattern of T cell development.

### Several chemokines interact with thymic fibroblasts in HS-dependent manner

Understanding how HS absence decreases the size of the thymus without blocking the development of any cell population is a difficult task. HS interacts with >300 extracellular molecules (30), and singling out any one of them as responsible for the phenotype is nearly impossible. We decided to take a candidate approached and started by testing the importance of the interaction of HS with the chemokines CCL19, CCL21, and CXCL12 that have well-characterized roles in directing thymocytes to different microenvironments (9, 13, 14). Binding of these chemokines to HS on thymic fibroblasts could establish localized fields and haptotactic gradients that could be important for the motility of various cells in the organ and their successful development.

**Figure 7:**
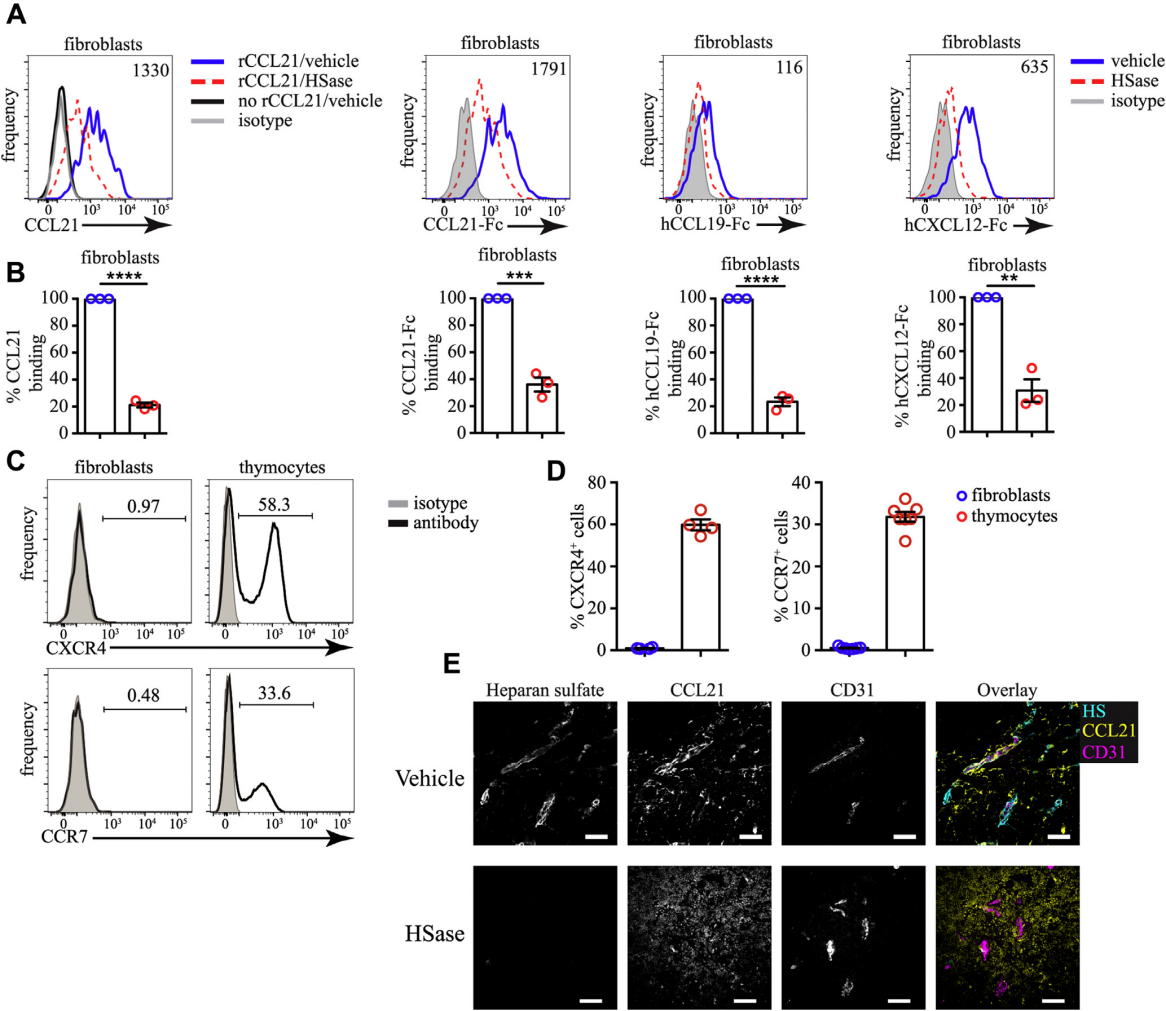
**CCL19, CCL21, and CXCL12 bind to thymic fibroblasts in an HS-dependent manner.***A*, flow cytometry plots of recombinant mouse CCL21 (rCCL21), mouse CCL21-Fc, human CCL21-Fc, and human CXCL12-Fc fusion proteins binding to thymic fibroblasts treated with HSase (*dashed*, *red*) or vehicle (*solid*, *blue*). The background is established by staining without rCCL21 (*solid*, *black*) and isotype (*gray*) controls. The numbers inside flow cytometry plots are the gMFI of chemokine binding without HSase treatment. *B*, quantification of the binding of rCCL21, CCL21-Fc, CCL19-Fc, and CXCL12-Fc to thymic fibroblasts after treatment with HSase or vehicle. The data are normalized, so that vehicle treatment is set to 100% in each experiment. *C*, flow cytometry staining for CXCR4 and CCR7 (*black*) on fibroblasts. CD45^-^ thymocytes serve as positive control, while isotype (*gray*) is negative control. The numbers inside flow cytometry plots indicate the proportion of positive cells. *D*, comparison of the percent of CXCR4^+^ (*left*) and CCR7^+^ (*right*) cells among fibroblasts and thymocytes. *E*, Immunofluorescent staining for HS, CCL21, and CD31 following treatment with a vehicle (*top*) or HSase digestion (*bottom*). Scale bars are 50 μm. All flow cytometry plots are representative of at least three independent experiments. Data in *B* and *D* are mean ± SEM. Each *dot* is an individual experiment. Statistical significance between treatment groups was determined with paired *t*-test, ∗∗*p* < 0.01, ∗∗∗*p* < 0.001, ∗∗∗∗*p* < 0.0001.

To find out if CCL19, CCL21, and CXCL12 can interact with thymic fibroblasts through HS, we used recombinant chemokines or Fc-fusion chemokines. We incubated single-cell suspensions from enzymatically digested thymuses with HSase to degrade HS and measured the binding of these proteins. Recombinant CCL21 bound well to thymic fibroblasts, but this binding was decreased ∼80% in the presence of HSase (Fig. 7, *A* and *B*). Importantly, HSase treatment was not toxic for fibroblasts or other cells in the thymus judged by viability staining with 4′,6-diamidino-2-phenylindole (DAPI) (Fig. S4). Identical data were obtained with CCL21-Fc fusion protein. CCL19-Fc and CXCL12-Fc could also bind well to thymic fibroblasts in an HS-dependent manner (Fig. 7, *A* and *B*). The fact that we used an identical detection method for the three fusion proteins allowed us to compare their relative strength of binding to HS. CCL21 had the strongest binding to fibroblasts, followed by CXCL12, and CCL19 had the weakest binding (Fig. 7, *A* and *B*). Importantly, neither fibroblasts nor ECs in the thymus expressed the receptors for CCL21/CCL19 (CCR7) or CXCL12 (CXCR4), while ∼60% of the thymocytes expressed CXCR4 and ∼30% expressed CCR7 (Fig. 7, *C* and *D*). Our data indicate that CCL19, CCL21, and CXCL12 can bind to HS on thymic fibroblasts.

To confirm this interaction *in situ*, we stained thymic sections with antibodies to HS and CCL21. CCL21 colocalized with HS around CD31^+^ blood vessels, but this colocalization was disrupted by HSase treatment of the section before the staining (Fig. 7*E*). CCL21 staining did not disappear, but became more diffuse and no longer accumulated around CD31^+^ structures. These data indicate that HS interacts with CCL21 around blood vessels.

### Disrupting HS–protein interactions interferes with cell migration in the thymus

To find out if the interactions of HS on thymic fibroblasts with chemokines are functionally relevant, we tested cell migration in thymic slices treated with HSase or Heparin, a highly negatively charged HS analog that can sequester interacting partners from HS (52). Thymic slices are thick (400 μm) preparations of living tissue that retain their three-dimensional structure and support motility and differentiation of thymocytes for several days (53). The migration of thymocytes in slices has been extensively characterized and found to be very similar to motility in intact thymuses (54). Another cell type that is known to migrate into thymic slices is DCs (55).

**Figure 8:**
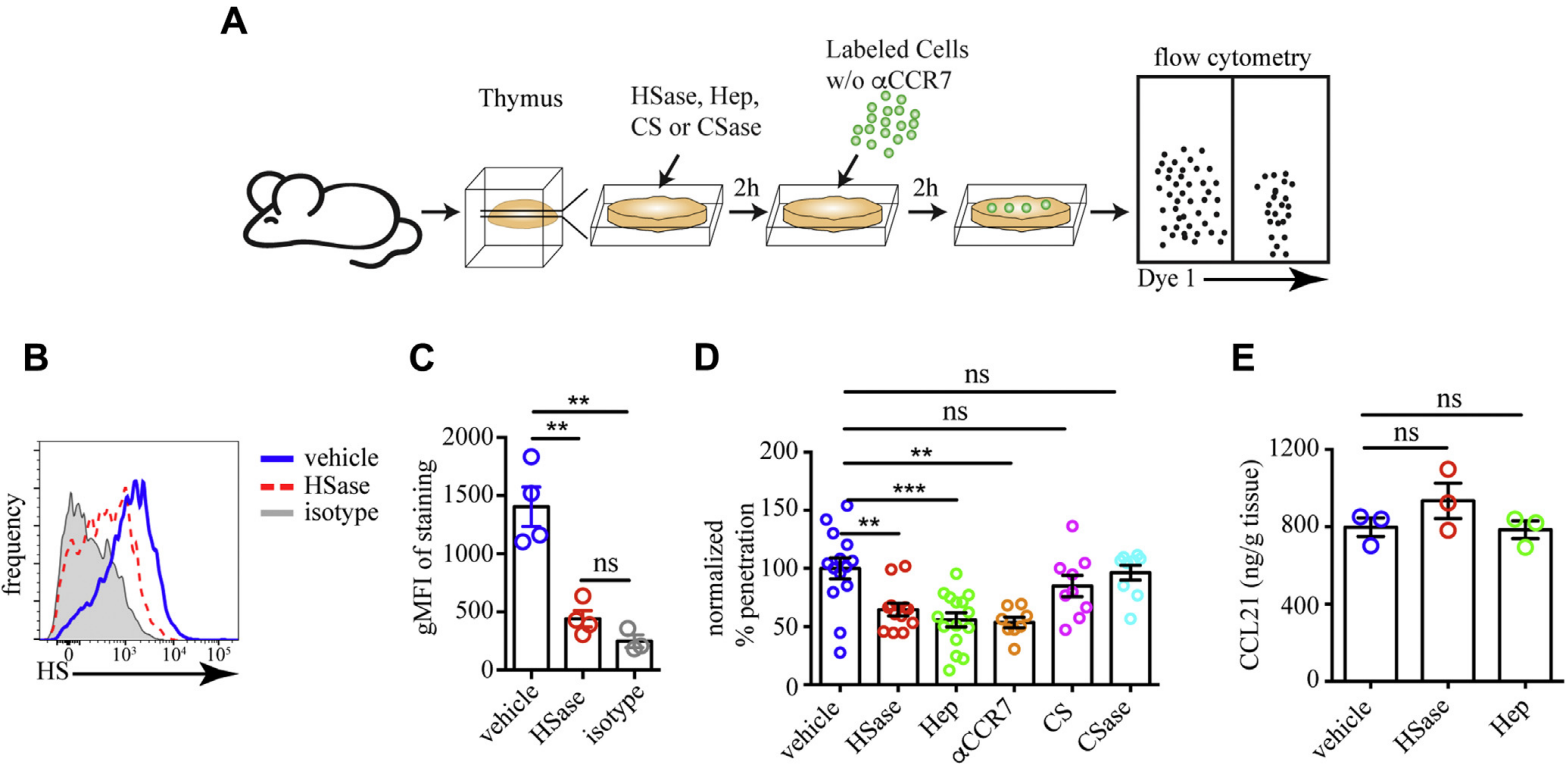
**Interference with HS impairs cell motility in thymic slices.***A*, scheme of the mature bone-marrow-derived dendritic cells (mBMDC) penetration into thymic slice experiment: Thymic slices (400 μm thick) were treated with HSase, Heparin (Hep), Chondroitinase ABC (CSase), Chondroitin sulfate (CS) or *left* untreated (control) and then overlaid with tdTomato-expressing mBMDCs that have been treated with anti-CCR7 antibody or not. After 2 hours, the cells that have failed to penetrate into the slice were washed off, and the percentage of fluorescent cells inside the slice was determined by flow cytometry. *B*, flow cytometry plots of HS expression on fibroblasts determined with the 10E4 antibody in slices that have been treated with HSase (*red*, *dashed*) or vehicle (*solid*, *blue*). Isotype serves as a negative control (*gray*). *C*, Comparison of the surface HS expression on fibroblasts from slices treated with HSase or vehicle. *D*, Comparison of the penetration of untreated mBMDC into thymic slices treated with vehicle, HSase, Heparin, Chondroitinase ABC, Chondroitin sulfate, and mBMDC treated with anti-CCR7 antibodies (αCCR7) into vehicle-treated slices. The data are normalized so that the average percentage of mBMDCs inside the slice with vehicle treatment in each experiment is set to 100%. *E*, concentration of CCL21 in slices treated with vehicle, HSase, or Heparin. The data in *C* and *E* are mean ± SEM from four or three independent experiments, respectively. Each *dot* is an individual mouse. The data in *D* are mean ± SEM from 3 to six independent experiments with three slices each. Each *dot* is an individual slice. Statistical significance in *C*, *D*, and *E* is determined by comparing all treatments to vehicle control by unpaired *t*-test ∗∗*p* < 0.01, ∗∗∗*p* < 0.001, ns, not significant.

We used LPS-treated mature bone-marrow-derived DCs (mBMDCs) as sensors for CCL19/21 chemokine fields in the tissue (56). These cells express CCR7, the receptor for CCL19/21, and migrate toward its ligands (56, 57). To determine if disruption of HS–chemokine interaction affects DC migration, we overlaid tdTomato-expressing mBMDCs on slices that have been treated with HSase or Heparin. After 2 h, we washed the surface of the slices of cells that have failed to migrate inside them and dissociated the slices. As a readout for the motility of the BMDCs, we determined the proportion of overlaid cells that have penetrated inside the slice by flow cytometry (Fig. 8*A*). We confirmed that HSase treatment greatly reduced the abundance of HS on fibroblasts in the slice (Fig. 8, *B* and *C*). The penetration of mBMDCs into the tissue was significantly impaired by HSase and Heparin treatment, but not by excess Chondroitin sulfate or Chondroitinase ABC (Fig. 8*D*), suggesting that chemokines interact specifically with HS but not all glycosaminoglycans. Moreover, pretreatment of mBMDCs with anti-CCR7 antibody impaired the penetration of the cells into the slice to the same extent as HSase or Heparin, confirming that CCR7 is the relevant receptor guiding the motility. Notably, the HSase or Heparin treatments did not change the abundance of CCL21, the primary ligand of CCR7 in the thymus (Fig. 8*E*), in line with the “chemokine cloud” hypothesis by Proudfoot and co-workers (58). Thus, interference with HS–chemokine interactions impairs the motility of mBMDCs in the thymus.

## Discussion

Here, we have investigated the distribution of HS in the thymus and its functional significance for thymus organogenesis and T cell development. We found that HS is most abundantly present on fibroblasts surrounding blood vessels. These cells can bind in an HS-dependent manner to several of the homeostatic chemokines that regulate thymocyte motility. The deletion of Ext1 in several stromal cell types led to multiple developmental abnormalities and death around E14.5. *In vitro* organ culture and embryonic thymus transplantations showed that the absence of HS interfered with the growth of the organ, but T cell development proceeded normally, albeit in reduced numbers. These defects could, at least partly, be explained by poor immobilization of several homeostatic chemokines and impaired interstitial motility within the thymus.

The phenotype of HS deficiency in the thymus is remarkably similar to the removal of mesenchymal cells from the thymus anlage—the organ failed to grow, but T cell development was not disturbed (22). This similarity suggests that one of the most critical roles of fibroblasts could be to supply HS required for the growth of the thymus during early life. This idea is supported by several studies that showed that the thymus can grow *in vitro* if thymic fibroblasts are chemically fixed or substituted by the fibroblast cell line 3T3 that expresses abundant HS (59, 60). The defective growth of mesenchyme-stripped thymuses has been attributed to the absence of growth factors such as Fgf7, Fgf10, Igf1, and Igf2 that fibroblasts secrete to promote the proliferation of TECs (20, 22). In our model of HS deficiency, the secretion of these growth factors should be preserved; however, they might be diffusing away too fast in the absence of the immobilization function of HS and unable to stimulate the proliferation of TECs efficiently.

Attributing the phenotype of HS deficiency in thymus to any secreted protein is a difficult task. At present, we do not have a technique to disrupt the HS binding only to a specific molecule. We have observed that HS binds to the homeostatic chemokines CCL19, CCL21, and CXCL12, and this interaction is physiologically important for the motility of the cells in the thymus. Thus, at least part of the effect of HS absence in the thymus can be attributed to the failure of immobilization of these chemokines and defective interstitial motility. Impaired directional migration can affect thymus development in several ways. 1) It can interfere with the attraction of bone-marrow-derived progenitors that rely on CCL19, CCL21, CXCL12, and CCL25 to enter the thymus (7–9), or 2) it can prevent thymocytes from progressing through development by impairing their access to microenvironments that support their differentiation. Impaired recruitment of progenitors results in a similar phenotype to HS deficiency in thymic fibroblast—smaller thymus in early life and regular pattern of T cell development (9). However, impaired progenitor influx cannot be the sole reason for the reduced size of the P-Ext1 thymus because it results in smaller thymus only during the embryonic period (9); in adult mice, the thymus size and cellularity are comparable with controls (7,8).

Disruption of the interaction of other secreted proteins with HS could also contribute to the defects in P-Ext1 thymuses. Impaired immobilization of fibroblast-derived growth factors supporting the proliferation of TECs such as Fgf7, Fgf10, Igf2 could result in failure of the thymus to grow due to lack of TEC expansion. All of these growth factors have been reported to interact with HS (61, 62). Moreover, mice deficient for the receptor for Fgf7 and Fgf10, FgfR2-IIIb, have smaller thymuses and normal T cell development (26). Finally, impaired function of growth factors for thymocytes, such as IL-7, could also contribute to the phenotype in P-Ext1 thymuses. IL-7 can interact with HS (33), and IL-7 deficiency results in a very small thymus with normal T cell developmental pattern (5). Thus, it is highly likely that the phenotype we observe is a sum of the disruption of many protein–HS interactions. In addition to the above examples, most secreted proteins with known roles in the development of the thymus, such as TGFβ, CCL25, and BMP4, can also interact with HS (63, 64, 65). This raises the possibility that HS is a global regulator of all intracellular interactions through secreted proteins.

Our analysis of cell migration revealed that HSase degradation and Heparin competition impair the motility of mBMDCs in thymic slices. While mBMDCs are not a normal constituent of the thymus, they express CCR7 and migrate to sources of CCR7 ligands (56). We used them purely as sensors for CCL19/21 gradients. The impaired penetration of mBMDCs into thymic tissue indicates disrupted CCL19/21 gradients and supports the idea that HS is essential for the creation of chemokine gradients in the thymus as demonstrated for lymphatic vessels (39). Mature CD4SP and CD8SP thymocytes and thymic DCs use the physiological CCL19/21 gradients to position themselves in the medulla, which is important for their selection and function, respectively (16).

Although HSase and Heparin treatment of thymic slices impaired mBMDC motility, the concentration of CCL21 was not reduced, which was expected because of the lack of washing steps. This result suggests that only a portion of the chemokines in a tissue is biologically active. The addition of Heparin, a highly active form of HS, or the creation of extra HS fragments by HSase digestion decreases this pool of active chemokines as evidenced by decreased mBMDC penetration into the slices. This result is consistent with the “chemokine cloud” hypothesis put forward by the late Amanda Proudfoot and co-workers, stating that the biologically active form of a chemokine is the HS-free one (58). Thus, both HSase and Heparin reduce the fraction of active chemokines by providing extra HS binding sites. Altogether our experiments have revealed that the amount of HS within a tissue needs to be carefully regulated as the absence of HS and excess HS can impair extracellular signaling molecules’ function.

The conditional deletion of HS in our studies was achieved with Pdgfrα^Cre^. However, this Cre transgene should delete Ext1 in most of the fibroblasts in the body, leading to embryonic lethality. Moreover, Cre activity is not restricted to fibroblasts because many TECs and some thymocytes also contained recombined loci (Fig. 3*B*). Other Cre lines currently used to study thymic fibroblasts include Wnt1^Cre^ and Sox10^Cre^ (17, 18). However, neither of them is specific for these cells. Recently, Wt1^Cre^ (66, 67) and Twist2^Cre^ (23) have been used successfully to study the functions of specific populations of fibroblasts in the body cavities and spleen and thymus, respectively. However, neither of them seem to be active in the whole fibroblast population under study. Thus, mouse lines, in which Cre activity is restricted to fibroblasts only and even to subpopulations of fibroblasts (*e.g.*, thymic fibroblasts), will greatly enhance our understanding of the function of these cells in various contexts.

## Experimental procedures

### Mice

C57BL/6Narl mouse was purchased from the National Laboratory Animal Center, NARLabs, Taipei, Taiwan, an AAALAC-accredited facility. Ext1^f/f^ (C57BL/6-Ext1^tm1Yama^) mice have been described (51). Pdgfrα^Cre^ (C57BL/6-Tg(Pdgfrα-Cre)1Clc/J, JAX stock#013148) (49) and R26-LSL-GFP (B6.Cg-Gt(ROSA)26Sor^tm6(CAG-ZsGreen1)Hze^/J, JAX stock#007906) (50) mice were purchased from The Jackson Laboratory. UBC-tdTomato mice were bred out from OT1 UBC-tdTomato (C57BL/6-Tg(TcrαTcrβ)1100Mjb Tg(UBC-tdTomato)1Narl/Narl) mice were purchased from the National Laboratory Animal Center, NARLabs, Taipei, Taiwan, and maintained by breeding homozygous mice. All mice were housed in the animal facility of National Yang-Ming University (NYMU) and used between 6 and 12 weeks of age. All animal experiments were approved by the Institutional Animal Care and Use Committee (IACUC) of NYMU.

### Cell isolation

Thymuses were cut into small pieces with a blade. Complete DMEM (cDMEM) medium containing high-glucose DMEM, 10% fetal calf serum, 1% Penicillin/Streptomycin, 1% L-Glutamine, 0.1% 2-Mercaptoethanol (all from Gibco) was added, and thymocytes were mechanically dissociated by pipetting. FTOCs were disrupted by gentle pipetting. The tissue pieces were collected and digested with cDMEM containing 0.2 mg/ml Collagenase P (Roche), 1.6 mg/ml Dispase (ThermoFisher), and 0.2 mg/ml DNase I (Roche) at 37 °C for 10–20 min. The cells were filtered through 70 μm Nylon mesh (Small Parts), washed with phosphate buffered saline (PBS), and resuspended in 3 ml PBS.

### Flow cytometry

Single-cell suspensions (0.5 – 2 x 10^6^ cells) from thymuses or FTOCs were blocked with 100 μl supernatant from 2.4G2 hybridoma (a kind gift by Dr Fang Liao, Academia Sinica, Taipei, Taiwan) and stained with fluorochrome- or biotin-labeled antibodies for 20 min on ice in 100 μl FACS buffer [PBS +0.5% BSA (HM Biological) + 1 mM EDTA (Merck) + 0.1% NaN_3_ (Sigma)]. The following antibodies were used: anti-mouse CD4-FITC (clone GK1.5), anti-mouse CD8α-APC/Fire750 (clone 53–6.7), anti-mouse CD25-PE (clone PC61), anti-mouse/human CD44-PE/cy7 (clone IM7), anti-mouse TCRβ-APC (clone H57–597), anti-mouse TCRγ/δ-PerCP/Cy5.5 (clone GL3), anti-mouse CD45-PerCP/Cy5.5 (clone 30-F11), anti-mouse CD326 (EpCAM)-APC/Cy7 (clone G8.8), anti-mouse Podoplanin (gp38)-PE/Cy7 (clone 8.1.1), anti-mouse PDGFRα-biotin (clone APA5), anti-mouse CCR7-PE (clone 4B12), anti-mouse CXCR4-PE (clone L276F12), anti-mouse F4/80-BV421 (clone BM8), anti-mouse CD11c-PE/cy7 (clone N418), anti-mouse I-A/I-E-APC/Cy7 (clone M5/114.15.2), anti-CD31-AF647 (clone 390) from BioLegend. After the staining, the cells were washed with FACS buffer, and if necessary, incubated for 20 more min with Streptavidin-PE (BioLegend). HS was stained with anti-Heparan sulfate antibody (clone F58–10E4, AMSBIO) and detected with donkey anti-mouse IgM-biotin (Jackson Immunoresearch) followed by Streptavidin-PE (BioLegend) or Streptavidin-DyLight488 (Jackson Immunoresearch). Alternatively, for detection of HS with 3G10 antibody, the single-cell suspension was treated with a mixture of Heparinase I + Heparinase II + Heparinase III (HSase) from *Bacteroides eggerthii* (NEB) at 0.5 U/ml each in DMEM (Gibco) for 1 h at 37˚C, 5% CO_2_ to reveal the epitope recognized by the antibody. After blocking with 24G2 supernatant and 2% rat serum (Jackson Immunoresearch), the cells were stained for 40 min in ice with the anti-HS antibody clone 3G10 (AMSBIO) or mouse IgG2b (Biolegend) as an isotype control. The staining was revealed with rat anti-mouse IgG2b-biotin (BioLegend) followed by Streptavidin-BV421 (BioLegend). The cells were then washed again in FACS buffer and resuspended in 300 μl FACS buffer. DAPI (ThermoFisher) at 3 μM or 0.5 μg/ml Propidium Iodide (Sigma) was added to exclude dead cells. The data were acquired on LSR Fortessa (BD Biosciences) flow cytometer running Diva 8 software and analyzed with FlowJo 10.6.2 (BD Biosciences).

### Immunofluorescent microscopy

Dissected thymus lobes from C57BL/6 were cleaned of connective tissue and fixed in 4% paraformaldehyde (Sigma) for 1 h at 4˚C, washed in PBS, submerged in 10% sucrose and then in 30% sucrose for 12 h each. The tissue was then frozen in Tissue-Tek OCT compound (Sakura Fintek) for cryostat sectioning. In some cases, the C57BL/6 thymuses and all transplanted thymuses from Pdgfrα^Cre^ X Ext1^f/f^ or control donors were freshly frozen in Tissue-Tek OCT compound without prior fixation. Tissue sections, 10–20 μm thick, were prepared with CryoStar NX50 (ThermoFisher) on SuperFrost PLUS (ThermoScientific) microscope slides, dried overnight, and stored at –80˚C until used. Unless otherwise stated, before staining, the sections were fixed with acetone (Sigma) at –20˚C for 10 min, air-dried, then blocked with 5% serum from the species in which the secondary antibody was raised in 24G2 supernatant (blocking buffer) for 2 h at room temperature. For staining HS, 100 μg/ml goat anti-mouse IgM Fab fragments (Jackson Immunoresearch) were also added for blocking endogenous IgM. The sections were washed four times with PBS and stained with primary antibodies overnight at 4˚C. After four washes in PBS, the sections were incubated with the secondary antibodies in blocking buffer for 2 h at room temperature. If necessary, after four more washes, the sections were incubated with fluorochrome-conjugated Streptavidin for two more hours at room temperature. Finally, the slides were stained with 3 μM DAPI (BioLegend) in PBS for 5 min and washed four times with PBS. The sections were mounted with 0.1% n-propyl gallate (Sigma) in glycerol (Sigma) and imaged with an AxioObserver 7 (Carl Zeiss) wide-field microscope. Image analysis was performed with Zen Blue 2.1 (Zeiss) and Imaris 8.0.2 (Bitplane). The following primary antibodies were used: rabbit anti-Laminin (Millipore), rabbit anti-CCL21 (R&D Systems), rabbit anti-PDGFRβ (clone 28E1, Cell Signaling), and anti-mouse CD31-AF647 (clone 390, BioLegend),. They were detected with anti-rabbit Cy3, or anti-rabbit AF647 (both from Jackson Immunoresearch). For HS staining, the primary antibody was mouse anti-heparan sulfate IgM antibody (clone F58-10E4, Amsbio) that was detected with donkey anti-mouse IgM-biotin antibody (Jackson Immunoresearch) followed by Streptavidin-Cy3 (BioLegend) or Streptavidin-DyLight488 (Jackson Immunoresearch). In some cases, sections from freshly frozen C57BL/6 thymuses were treated with 100 μl of HSase at 1 U/ml each in Heparinase buffer (NEB) for 1 h at 37˚C before the acetone fixation and then stained for HS, CCL21, and CD31.

### Timed pregnancies and embryonic thymus harvesting

Male Pdgfrα^Cre^ X Ext1^f/+^ mice were mated with female Ext1^f/f^ mice and separated the next day (E0.5). At day 14.5, after conception, the pregnant dam was sacrificed, the embryos were separated from the surrounding tissues, and the thymuses were collected under a dissecting microscope (Leica S8 APO) in PBS. Embryos with tissue abnormalities were considered Pdgfrα^Cre^ X Ext1^f/f^, while visually healthy embryos were considered controls (Pdgfrα^Cre^ X Ext1^f/+^, Ext1^f/f^, or Ext1^f/+^). The genotypes were confirmed by PCR on DNA extracted from a piece of the liver of each embryo. Images of the embryos, E14.5 thymuses, and day 7 FTOCs were taken with HD Lite 1080p camera (KLBiotech, Taiwan). The lobes of the thymus were separated and used for FTOCs or engrafted under the kidney capsule of a C57BL/6 mouse.

### FTOCs

Control or Pdgfrα^Cre^ X Ext1^f/f^ fetal thymuses were separated into individual lobes under a dissecting microscope and cultured in a 6-well plate on 0.4 μm pore size Cell Culture Inserts (Falcon) with 1 ml cDMEM medium under them at 37 °C in a 5% CO_2_ incubator. The 6-well plate was put in the container with 5 ml of sterile dH_2_O inside to prevent the medium from evaporation, and a small hole was made on the lid for gas equilibration. After 7 days, the lobes from the same genotype were pooled together and mechanically (for thymocyte analysis) or enzymatically (for stromal cell analysis) dissociated, and analyzed by flow cytometry. Samples with viability less than 50% were excluded from analysis (two control and three P-Ext1).

### Embryonic thymus transplantations

The recipient C57BL/6 mice were anesthetized by i.p. injection with 120 mg/kg Ketamine hydrochloride (Toronto Research Chemicals) and 12 mg/kg Xylazine hydrochloride (Sigma). The left flank was shaved and disinfected, and incisions were made on the skin above the kidney, the muscle layer, and the peritoneum. The left kidney was exposed through the incision, and two embryonic thymus lobes were implanted under the kidney capsule. The organ was repositioned into the abdominal cavity, the muscle layer was closed with sutures, and the skin was stapled. Rimadyl (Carprofen, Zoetis) was given s.c. once at a dose of 5 mg/kg to relieve pain, and mice were given water with 0.5 mg/ml Sulfadiazine +0.1 mg/ml Trimethoprim (Trimerin, Center Labs, Taiwan) for 7 days. The transplants were allowed to grow for 6 weeks, and the recipients were sacrificed for analysis.

### Recombinant proteins

Mouse CCL21 sequence was amplified from thymus cDNA by PCR with the following primers F1: CTG**GCTAGC**ATGGCTCAGATGATGACTCTGAG, R1: GTC**GGATCC**TCTTGAGGG CTGTGTC and cloned into pJET1.2 (CloneJET PCR Cloning Kit, Thermo Fisher). The sequence was then cloned into the NheI + BamHI (both from NEB) digested ELC-Fc vector, a kind gift from Dr Timothy Springer (Addgene plasmid # 8636), replacing the original CCL19 sequence and fusing in-frame with a mutated version of human IgG1 Fc sequence (68). The recombinant fusion protein was produced in Expi293 cells (ThermoFisher) following the manufacturer's recommendations and purified using AktaPrime Plus (GE) with a HiTrap Protein A HP column (GE). The buffer was exchanged to phosphate buffer with a Vivaspin 20 column (molecular cutoff 10 kD), and the protein was frozen in 10% Glycerol and stored at –80˚C until used. Human CCL19 was purified in the same way as CCL21, but the original ELC-Fc vector was used. Human CXCL12 was purchased from Sinobiological. Recombinant mouse CCL21 was from BioLegend.

### HS binding assay

Enzymatically digested thymus cell suspensions were enriched for stromal cells by density centrifugation. Briefly, 3 ml single-cell suspension in PBS was underlaid with 57% Percoll PLUS (GE) in PBS and spun for 20 min at 1800 rpm at 4 °C without brake. The cells at the interface were collected and transferred into a new tube, washed with PBS, and used for staining. Typically, 10^6^ cells were used in a single test. First, the cells were incubated with 6 U/ml Heparin (Sigma) for 10 min in ice in PBS to remove all HS-bound proteins, or with 100 μl of a mixture of *B. eggerthii* HSases (NEB) at 0.5 U/ml each in PBS for 1 h at 37˚C to digest HS, or with PBS only. After washing with FACS buffer, the cells were treated with 4 μg/ml recombinant CCL21 or 10 μg/ml of the different Fc fusion proteins for 1 h on ice. CCL21 was detected with anti-CCL21 antibody (LifeSpan) followed by anti-rabbit-PE (Jackson Immunoresearch). The Fc fusion proteins were detected with anti-human IgG-biotin (Jackson Immunoresearch) preincubated with 2% rat serum (Jackson Immunoresearch) followed by Streptavidin-PE (BioLegend). The cells were also stained for HS (10E4), CD45, EpCAM, and gp38, as described in the Flow Cytometry section.

### Thymic slices preparation

Thymic slices were prepared essentially as described (53). Briefly, the thymus was dissected, the two lobes were separated and carefully cleaned out of connective tissue. Individual lobes were embedded in 4% low-melting-point agarose (GTG-NuSieve, Lonza) in HBSS (Gibco). The resulting block was trimmed of excess agarose, glued onto the stage of Vibratome 1000S (Leica), submerged in ice-cold PBS, and cut into 400 μm thick slices. The slices were put on 0.4 μm pore size Cell Culture Inserts (Falcon) in a 6-well tissue culture plate (Falcon) containing 1 ml of cDMEM under the inserts. The slices were treated in the following way: 1) vehicle control—the slices were overlaid with 10 μl cDMEM; 2) HSase—the slices were overlaid with 10 μl of a mixture of *B. eggerthii* HSases (NEB) at 2 U/ml each in DMEM and incubated in a 5% CO_2_ incubator at 37˚C for 2 h to eliminate HS from the slices; 3) Heparin—the slices were soaked in 0.1 ml of cDMEM containing 6 U/ml Heparin (Sigma) in a 5% CO_2_ incubator for 2 h at 37˚C to disrupt HS–protein interaction; 4) Chondroitinase ABC—the slices were overlaid with 10 μl of Chondroitinase ABC from *Proteus vulgaris* (Sigma) at 1 U/ml in DMEM and incubated in a 5% CO_2_ incubator at 37˚C for 2 h to eliminate chondroitin sulfate from the slices; 5) Chondroitin sulfate—the slices were soaked in 0.1 ml of cDMEM containing 18 μg/ml Chondroitin sulfate (Sigma), which is equivalent concentration to the Heparin treatment above, in a 5% CO_2_ incubator for 2 h at 37˚C as a negative control for Heparin treatment.

### Bone-marrow-derived dendritic cells (BMDCs)

BMDCs were cultured as described (69). Briefly, the femurs and tibiae of UBC-tdTomato mice were rinsed with 70% ethanol and then with sterile PBS. The bone marrow was flushed with sterile RPMI (Gibco), pipetted several times to break down the clumps, and filtered through 70 μm Nylon mesh. Two million bone marrow cells were seeded in 10 cm Petri dishes (Alpha, Taiwan) in complete RPMI containing 10% fetal bovine serum, 1% Penicillin/Streptomycin, 1% L-Glutamine, 0.1% 2-Mercaptoethanol, and 20 ng/ml recombinant mouse GM-CSF (BioLegend) and cultured in a 37˚C incubator with 5% CO_2_. Ten milliliter of fresh medium with 20 ng/ml mouse GM-CSF was added on day 3. Half of the medium was replaced with fresh GM-CSF-containing medium on days 6 and 8. The nonadherent and loosely adherent cells were harvested by gentle pipetting on day 10 and labeled as immature BMDC. Mature BMDCs (mBMDCs) were produced from the immature BMDCs by treatment with 200 ng/ml LPS (Invivogen) overnight. The nonadherent and poorly adherent cells were harvested and labeled as mBMDC

### BMDC penetration assay

UBC-tdTomato^+^ mBMDCs were harvested, washed in PBS, and resuspended in cDMEM at 5 x 10^5^ cells/10 μl. For the CCR7 blocking experiments, 2 x 10^6^ mBMDCs in 100 μl cDMEM were treated with 15 μg anti-CCR7 antibody clone 4B12 (ThermoFisher) in a 5% CO_2_ incubator for 2 h at 37˚C as described (70). The cells were washed with cDMEM and resuspended in cDMEM at 5 x 10^5^ cells/10 μl. Thymic slices that have been treated with vehicle, HSase, Heparin, Chondroitinase ABC, or Chondroitin sulfate were transferred to fresh Cell Culture Inserts in 6-well plates containing 1 ml cDMEM. Each slice was carefully overlaid with 10 μl (∼5 x 10^5^) mBMDCs and kept for 2 h in a 5% CO_2_ incubator at 37˚C. Anti-CCR7 treated mBMDCs were overlaid on vehicle-treated slices and incubated at the same conditions as other mBMDCs. After the end of the incubation, each slice was gently washed with cDMEM to remove cells that have failed to penetrate inside it. Each slice was transferred to an Eppendorf tube and enzymatically digested with 500 μl of 0.2 mg/ml Collagenase P (Roche) + 0.2 mg/ml DNase I (Roche) in DMEM for 20 min at 37˚C with frequent agitation. The cell suspension was filtered through 70 μm Nylon mesh into FACS tubes (Falcon) to remove the remaining agarose pieces, washed with FACS buffer, and resuspended in 300 μl FACS buffer containing 3 μM DAPI (ThermoFisher). The data were acquired on LSR Fortessa (BD Biosciences) flow cytometer running Diva 8 software and analyzed with FlowJo 10.6.2 (BD Biosciences).

### Measurement of CCL21 concentration in thymic slices

Thymic slices treated with vehicle, 1 U/ml HSase, or 6 U/ml Heparin, as described above, were carefully freed from the agarose, weighed, and homogenized in T-PER buffer (ThermoFisher) supplemented with cOmplete Mini protease inhibitors (Roche). The slices were homogenized by 100 strokes in Dounce homogenizer (Wheaton) and incubated for an additional 1 h in ice. The lysate was centrifuged for 5 min at 10,000 *g* at 4˚C and stored at –80 °C until used. The ELISA for CCL21 was done with DuoSet mouse CCL21/6Ckine kit (R&D Systems) according to the manufacturer’s recommendations. The concentrations determined by ELISA were normalized to the weight of the tissue.

### Statistical analysis

Prism 6.0 (GraphPad) was used for creating graphs and statistical analyses. A paired *t*-test was used when comparing protein binding to fibroblasts with or without HSase treatment in Figure 7. In all other cases, an unpaired two-tailed *t*-test was used when comparing two groups, and one-way ANOVA with Tukey posttest was applied when more than two groups were compared. All data are presented as mean ± SEM. A *p*-value lower than 0.05 is considered statistically significant.

## Data availability

All the data are contained within the article.

## Supporting information

This article contains supporting information.

## Conflict of interest

The authors declare that the research was conducted in the absence of any commercial or financial relationships that could be construed as a potential conflict of interest.

## References

[1] Radtke F., Wilson A., Stark G., Bauer M., van Meerwijk J., MacDonald H.R., Aguet M. (1999). Deficient T cell fate specification in mice with an induced inactivation of Notch1. Immunity.

[2] Yuan J.S., Kousis P.C., Suliman S., Visan I., Guidos C.J. (2010). Functions of Notch signaling in the immune system: Consensus and Controversies. Annu. Rev. Immunol..

[3] Klein L., Kyewski B., Allen P.M., Hogquist K.A. (2014). Positive and negative selection of the T cell repertoire: What thymocytes see (and don’t see). Nat. Rev. Immunol..

[4] Breed E.R., Lee S.T., Hogquist K.A. (2017). Directing T cell fate: How thymic antigen presenting cells coordinate thymocyte selection. Semin. Cell. Dev. Biol..

[5] Freeden-Jeffry, von U., Vieira P., Lucian L.A., McNeil T., Burdach S.E., Murray R. (1995). Lymphopenia in interleukin (IL)-7 gene-deleted mice identifies IL-7 as a nonredundant cytokine. J. Exp. Med..

[6] Hu Z., Lancaster J.N., Ehrlich L.I.R. (2015). The contribution of chemokines and migration to the induction of central tolerance in the thymus. Front Immunol..

[7] Krueger A., Willenzon S., Lyszkiewicz M., Kremmer E., Förster R. (2010). CC chemokine receptor 7 and 9 double-deficient hematopoietic progenitors are severely impaired in seeding the adult thymus. Blood.

[8] Zlotoff D.A., Sambandam A., Logan T.D., Bell J.J., Schwarz B.A., Bhandoola A. (2010). CCR7 and CCR9 together recruit hematopoietic progenitors to the adult thymus. Blood.

[9] Calderón L., Boehm T. (2011). Three chemokine receptors cooperatively regulate homing of hematopoietic progenitors to the embryonic mouse thymus. Proc. Natl. Acad. Sci..

[10] Porritt H.E., Gordon K., Petrie H.T. (2003). Kinetics of Steady-state differentiation and Mapping of intrathymic-signaling Environments by Stem cell transplantation in Nonirradiated mice. J. Exp. Med..

[11] Benz C., Heinzel K., Bleul C.C. (2004). Homing of immature thymocytes to the subcapsular microenvironment within the thymus is not an absolute requirement for T?cell development. Eur. J. Immunol..

[12] Halkias J., Melichar H.J., Taylor K.T., Ross J.O., Yen B., Cooper S.B., Winoto A., Robey E.A. (2013). Opposing chemokine gradients control human thymocyte migration *in situ*. J. Clin. Invest..

[13] Kadakia T., Tai X., Kruhlak M., Wisniewski J., Hwang I.-Y., Roy S., Guinter T.I., Alag A., Kehrl J.H., Zhuang Y., Singer A. (2019). E-protein-regulated expression of CXCR4 adheres preselection thymocytes to the thymic cortex. J. Exp. Med..

[14] Ueno T., Saito F., Gray D.H.D., Kuse S., Hieshima K., Nakano H., Kakiuchi T., Lipp M., Boyd R.L., Takahama Y. (2004). CCR7 signals are essential for cortex-medulla migration of developing thymocytes. J. Exp. Med..

[15] Hu Z., Lancaster J.N., Sasiponganan C., Ehrlich L.I.R. (2015). CCR4 promotes medullary entry and thymocyte-dendritic cell interactions required for central tolerance. J. Exp. Med..

[16] Kurobe H., Liu C., Ueno T., Saito F., Ohigashi I., Seach N., Arakaki R., Hayashi Y., Kitagawa T., Lipp M., Boyd R.L., Takahama Y. (2006). CCR7-Dependent cortex-to-medulla migration of positively selected thymocytes is essential for establishing central tolerance. Immunity.

[17] Muller S.M., Stolt C.C., Terszowski G., Blum C., Amagai T., Kessaris N., Iannarelli P., Richardson W.D., Wegner M., Rodewald H.R. (2008). Neural crest origin of perivascular mesenchyme in the adult thymus. J. Immunol..

[18] Foster K., Sheridan J., Veiga-Fernandes H., Roderick K., Pachnis V., Adams R., Blackburn C., Kioussis D., Coles M. (2008). Contribution of neural crest-derived cells in the embryonic and adult thymus. J. Immunol..

[19] Odaka C. (2009). Localization of mesenchymal cells in adult mouse thymus: Their abnormal distribution in mice with disorganization of thymic medullary epithelium. J. Histochem. Cytochem..

[20] Jenkinson W.E., Jenkinson E.J., Anderson G. (2003). Differential requirement for mesenchyme in the proliferation and maturation of thymic epithelial progenitors. J. Exp. Med..

[21] Sitnik K.M., Kotarsky K., White A.J., Jenkinson W.E., Anderson G., Agace W.W. (2012). Mesenchymal cells regulate retinoic acid receptor-dependent cortical thymic epithelial cell homeostasis. J. Immunol..

[22] Jenkinson W.E., Rossi S.W., Parnell S.M., Jenkinson E.J., Anderson G. (2007). PDGFRα-expressing mesenchyme regulates thymus growth and the availability of intrathymic niches. Blood.

[23] Nitta T., Tsutsumi M., Nitta S., Muro R., Suzuki E.C., Nakano K., Tomofuji Y., Sawa S., Okamura T., Penninger J.M., Takayanagi H. (2020). Fibroblasts as a source of self-antigens for central immune tolerance. Nature.

[24] Zachariah M.A., Cyster J.G. (2010). Neural crest-derived pericytes promote egress of mature thymocytes at the corticomedullary junction. Science.

[25] Sun L., Sun C., Liang Z., Li H., Chen L., Luo H., Zhang H., Ding P., Sun X., Qin Z., Zhao Y. (2015). FSP1(+) fibroblast subpopulation is essential for the maintenance and regeneration of medullary thymic epithelial cells. Nature.

[26] Revest J.M., Suniara R.K., Kerr K., Owen J.J., Dickson C. (2001). Development of the thymus requires signaling through the fibroblast growth factor receptor R2-IIIb. J. Immunol..

[27] Gallagher J. (2015). Fell-muir Lecture: Heparan sulphate and the art of cell regulation: A polymer chain conducts the protein orchestra. Int. J. Exp. Pathol..

[28] McCormick C., Duncan G., Goutsos K.T., Tufaro F. (2000). The putative tumor suppressors EXT1 and EXT2 form a stable complex that accumulates in the Golgi apparatus and catalyzes the synthesis of heparan sulfate. Proc. Natl. Acad. Sci. U. S. A..

[29] Lin X., Wei G., Shi Z., Dryer L., Esko J.D., Wells D.E., Matzuk M.M. (2000). Disruption of gastrulation and heparan sulfate biosynthesis in EXT1-deficient mice. Dev. Biol..

[30] Xu D., Esko J.D. (2014). Demystifying heparan sulfate–protein interactions. Annu. Rev. Biochem..

[31] Bao X., Moseman E.A., Saito H., Petryanik B., Thiriot A., Hatakeyama S., Ito Y., Kawashima H., Yamaguchi Y., Lowe J.B., Andrian Von, U.H., Fukuda M. (2010). Endothelial heparan sulfate ControlsChemokine presentation in Recruitmentof lymphocytes and dendritic cells to lymph nodes. Immunity.

[32] Amara A., Lorthioir O., Valenzuela A., Magerus A., Thelen M., Montes M., Virelizier J.L., Delepierre M., Baleux F., Lortat-Jacob H., Arenzana-Seisdedos F. (1999). Stromal cell-derived factor-1alpha associates with heparan sulfates through the first beta-strand of the chemokine. J. Biol. Chem..

[33] Banwell C.M., Partington K.M., Jenkinson E.J., Anderson G. (2000). Studies on the role of IL-7 presentation by mesenchymal fibroblasts during early thymocyte development. Eur. J. Immunol..

[34] Wrenshall L.E., Platt J.L. (1999). Regulation of T cell homeostasis by heparan sulfate-bound IL-2. J. Immunol..

[35] Gordts P.L.S.M., Foley E.M., Lawrence R., Sinha R., Lameda-Diaz C., Deng L., Nock R., Glass C.K., Erbilgin A., Lusis A.J., Witztum J.L., Esko J.D. (2014). Reducing macrophage proteoglycan sulfation increases atherosclerosis and obesity through enhanced type I Interferon signaling. Cell Metab..

[36] Massena S., Christoffersson G., Hjertström E., Zcharia E., Vlodavsky I., Ausmees N., Rolny C., Li J.-P., Phillipson M. (2010). A chemotactic gradient sequestered on endothelial heparan sulfate induces directional intraluminal crawling of neutrophils. Blood.

[37] Wang L., Fuster M., Sriramarao P., Esko J.D. (2005). Endothelial heparan sulfate deficiency impairs L-selectin- and chemokine-mediated neutrophil trafficking during inflammatory responses. Nat. Immunol..

[38] Tsuboi K., Hirakawa J., Seki E., Imai Y., Yamaguchi Y., Fukuda M., Kawashima H. (2013). Role of high endothelial Venule-expressed heparan sulfate in chemokine presentation and lymphocyte homing. J. Immunol..

[39] Weber M., Hauschild R., Schwarz J., Moussion C., de Vries I., Legler D.F., Luther S.A., Bollenbach T., Sixt M. (2013). Interstitial dendritic cell guidance by haptotactic chemokine gradients. Science.

[40] Yin X., Johns S.C., Kim D., Mikulski Z., Salanga C.L., Handel T.M., Macal M., Zúñiga E.I., Fuster M.M. (2014). Lymphatic specific disruption in the fine structure of heparan sulfate inhibits dendritic cell traffic and functional T cell responses in the lymph node. J. Immunol..

[41] Reijmers R.M., Vondenhoff M.F.R., Roozendaal R., Kuil A., Li J.-P., Spaargaren M., Pals S.T., Mebius R.E. (2010). Impaired lymphoid organ development in mice lacking the heparan sulfate modifying enzyme glucuronyl C5-epimerase. J. Immunol..

[42] Reijmers R.M., Groen R.W.J., Kuil A., Weijer K., Kimberley F.C., Medema J.P., van Kuppevelt T.H., Li J.-P., Spaargaren M., Pals S.T. (2011). Disruption of heparan sulfate proteoglycan conformation perturbs B-cell maturation and APRIL-mediated plasma cell survival. Blood.

[43] Garner O.B., Yamaguchi Y., Esko J.D., Videm V. (2008). Small changes in lymphocyte development and activation in mice through tissue-specific alteration of heparan sulphate. Immunology.

[44] Volpi S., Yamazaki Y., Brauer P.M., van Rooijen E., Hayashida A., Slavotinek A., Sun Kuehn H., Di Rocco M., Rivolta C., Bortolomai I., Du L., Felgentreff K., Ott de Bruin L., Hayashida K., Freedman G. (2017). EXTL3 mutations cause skeletal dysplasia, immune deficiency, and developmental delay. J. Exp. Med..

[45] Oud M.M., Tuijnenburg P., Hempel M., van Vlies N., Ren Z., Ferdinandusse S., Jansen M.H., Santer R., Johannsen J., Bacchelli C., Alders M., Li R., Davies R., Dupuis L., Cale C.M. (2017). Mutations in EXTL3 cause Neuro-immuno-skeletal dysplasia Syndrome. Am. J. Hum. Genet..

[46] David G., Bai X.M., Van Der Schueren B., Cassiman J.-J., Van Den Berghe H. (1992). Developmental changes in heparan sulfate expression: In situ detection with mAbs. J. Cell Biol..

[47] Hohenester E., Yurchenco P.D. (2014). Laminins in basement membrane assembly. Cell Adhes. Migration.

[48] Sitnik K.M., Wendland K., Weishaupt H., Uronen-Hansson H., White A.J., Anderson G., Kotarsky K., Agace W.W. (2016). Context-dependent development of lymphoid stroma from adult CD34(+) Adventitial progenitors. Cell Rep..

[49] Roesch K., Jadhav A.P., Trimarchi J.M., Stadler M.B., Roska B., Sun B.B., Cepko C.L. (2008). The transcriptome of retinal Müller glial cells. J. Comp. Neurol..

[50] Madisen L., Zwingman T.A., Sunkin S.M., Oh S.W., Zariwala H.A., Gu H., Ng L.L., Palmiter R.D., Hawrylycz M.J., Jones A.R., Lein E.S., Zeng H. (2009). A robust and high-throughput Cre reporting and characterization system for the whole mouse brain. Nat. Neurosci..

[51] Inatani M., Irie F., Plump A.S., Tessier-Lavigne M., Yamaguchi Y. (2003). Mammalian brain morphogenesis and midline axon guidance require heparan sulfate. Science.

[52] Mulloy B., Forster M.J. (2000). Conformation and dynamics of heparin and heparan sulfate. Glycobiology.

[53] Zhou T.-A., Hsu C.-L., Dzhagalov I.L. (2020). Testing the efficiency and Kinetics of negative selection using thymic slices. Methods Mol. Biol..

[54] Le Borgne M., Ladi E., Dzhagalov I.L., Herzmark P., Liao Y.F., Chakraborty A.K., Robey E.A. (2009). The impact of negative selection on thymocyte migration in the medulla. Nat. Immunol..

[55] Weist B.M., Kurd N., Boussier J., Chan S.W., Robey E.A. (2015). Thymic regulatory T cell niche size is dictated by limiting IL-2 from antigen-bearing dendritic cells and feedback competition. Nat. Immunol..

[56] Schumann K., Lämmermann T., Bruckner M., Legler D.F., Polleux J., Spatz J.P., Schuler G., Förster R., Lutz M.B., Sorokin L., Sixt M. (2010). Immobilized chemokine fields and soluble chemokine gradients cooperatively Shape migration patterns of dendritic cells. Immunity.

[57] Sallusto F., Schaerli P., Loetscher P., Schaniel C., Lenig D., Mackay C.R., Qin S., Lanzavecchia A. (1998). Rapid and coordinated switch in chemokine receptor expression during dendritic cell maturation. Eur. J. Immunol..

[58] Graham G.J., Handel T.M., Proudfoot A.E.I. (2019). Leukocyte Adhesion: Reconceptualizing chemokine presentation by glycosaminoglycans. Trends Immunol..

[59] Anderson G., Jenkinson E.J., Moore N.C., Owen J.J. (1993). MHC class II-positive epithelium and mesenchyme cells are both required for T-cell development in the thymus. Nature.

[60] Anderson G., Anderson K.L., Tchilian E.Z., Owen J.J.T., Jenkinson E.J. (1997). Fibroblast dependency during early thymocyte development maps to the CD25+ CD44+ stage and involves interactions with fibroblast matrix molecules. Eur. J. Immunol..

[61] Zhang F., Zheng L., Cheng S., Peng Y., Fu L., Zhang X., Linhardt R.J. (2019). Comparison of the interactions of different growth factors and glycosaminoglycans. Molecules.

[62] Lund J., Søndergaard M.T., Conover C.A., Overgaard M.T. (2014). Heparin-binding mechanism of the IGF2/IGF-binding protein 2 complex. J. Mol. Endocrinol..

[63] Lyon M., Rushton G., Gallagher J.T. (1997). The interaction of the Transforming growth factor-βs with heparin/heparan sulfate is Isoform-specific. J. Biol. Chem..

[64] de Paz J.L., Moseman E.A., Noti C., Polito L., Andrian Von, U.H., Seeberger P.H. (2007). Profiling heparin–chemokine interactions using Synthetic tools. ACS Chem. Biol..

[65] Ohkawara B., Iemura S.-I., Dijke ten, P., Ueno N. (2002). Action range of BMP is defined by its N-terminal basic amino acid core. Curr. Biol..

[66] Buechler M.B., Kim K.-W., Onufer E.J., Williams J.W., Little C.C., Dominguez C.X., Li Q., Sandoval W., Cooper J.E., Harris C.A., Junttila M.R., Randolph G.J., Turley S.J. (2019). A stromal niche defined by expression of the Transcription factor WT1 Mediates Programming and homeostasis of cavity-Resident macrophages. Immunity.

[67] Bellomo A., Mondor I., Spinelli L., Lagueyrie M., Stewart B.J., Brouilly N., Malissen B., Clatworthy M.R., Bajénoff M. (2020). Reticular fibroblasts expressing the Transcription factor WT1 define a stromal niche that maintains and Replenishes Splenic red Pulp macrophages. Immunity.

[68] Manjunath N., Shankar P., Wan J., Weninger W., Crowley M.A., Hieshima K., Springer T.A., Fan X., Shen H., Lieberman J., Andrian, von U.H. (2001). Effector differentiation is not prerequisite for generation of memory cytotoxic T lymphocytes. J. Clin. Invest..

[69] Lutz M.B., Kukutsch N., Ogilvie A.L., Rössner S., Koch F., Romani N., Schuler G. (1999). An advanced culture method for generating large quantities of highly pure dendritic cells from mouse bone marrow. J. Immunol. Methods.

[70] Peske J.D., Thompson E.D., Gemta L., Baylis R.A., Fu Y.-X., Engelhard V.H. (2015). Effector lymphocyte-induced lymph node-like vasculature enables naive T-cell entry into tumours and enhanced anti-tumour immunity. Nat. Commun..

