# Supplementary material for: Heparan sulfate is essential for thymus growth
Source: J Biol Chem. 2021 Feb 15;296:100419. doi: 10.1016/j.jbc.2021.100419 (PMC7974028; doi:10.1016/j.jbc.2021.100419)
Supplement: Figures S1–S4 [file mmc1.pdf]

Supporting information for:

Heparan sulfate is essential for thymus growth

Hsuan-Po Hsu[1], Yun-Tzu Chen[1], Yu-Ying Chen[1], Chih-Yu Lin[1], Po-Yu Chen[1], Shio-Yi Liao[1], Ciara C. Lim[1], Yu Yamaguchi[2], Chia-Lin Hsu[1], Ivan L. Dzhagalov[1*]

[1]Institute of Microbiology and Immunology, National Yang-Ming University, Taipei, Taiwan

[2]Sanford Burnham Prebys Medical Discovery Institute, La Jolla, CA, USA

[*]Corresponding Author: Ivan L. Dzhagalov

e-mail: ivan.dzhagalov@ym.edu.tw

Running title: Heparan sulfate is essential for thymus growth

Supporting information content:

Figure S1 Gating strategy for identification of stromal cells in thymus, lymph nodes and spleen.

Figure S2 Expression of HS in the thymus determined with 3G10 antibody after HSase digestion.

Figure S3 Expression of the glycosyltransferases Ext1 and Ext2 in cells in the thymus and fibroblasts in different organs.

Figure S4 HSase treatment does not affect the viability of cells in the thymus, including fibroblasts.

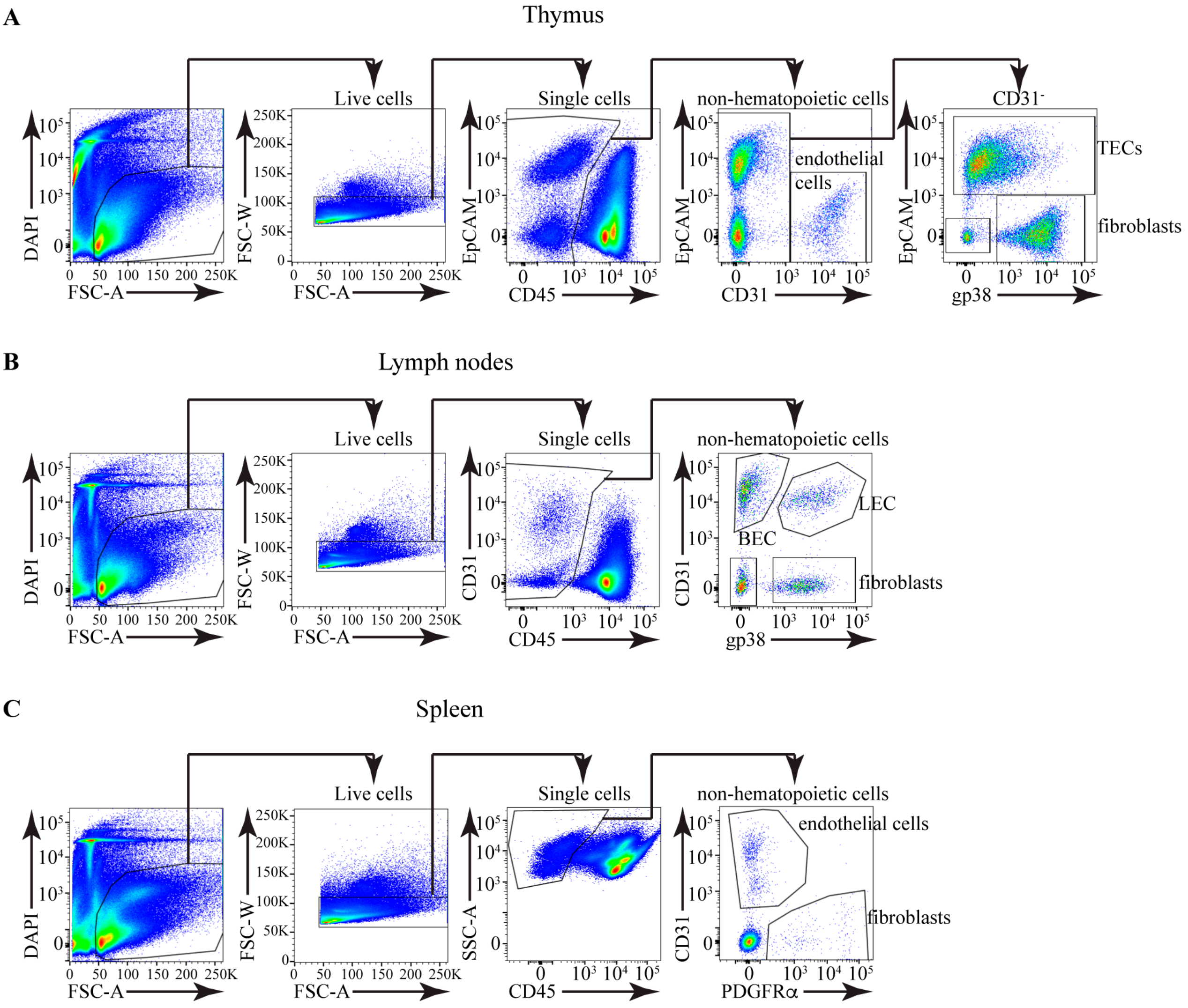

Figure S1: Gating strategy for identification of stromal cells in thymus, lymph nodes and spleen. A. In the thymus fibroblasts are identified as CD45$^-$CD31$^-$EpCAM$^-$gp38$^+$ cells; thymic epithelial cells are CD45$^-$CD31$^-$EpCAM$^+$ cells; endothelial cells are CD45$^-$CD31$^+$ cells. **B.** In the lymph node fibroblasts are identified as CD45$^-$CD31$^-$gp38$^+$ cells; blood endothelial cells (BECs) are CD45$^-$CD31$^+$gp38$^-$ cells; lymphatic endothelial cells (LECs) are CD45$^-$CD31$^+$gp38$^+$ cells. **C.** In the spleen fibroblasts are identified as CD45$^-$CD31$^-$PDGFRα$^+$ cells; endothelial cells are CD45$^-$CD31$^+$PDGFRα$^-$ cells.

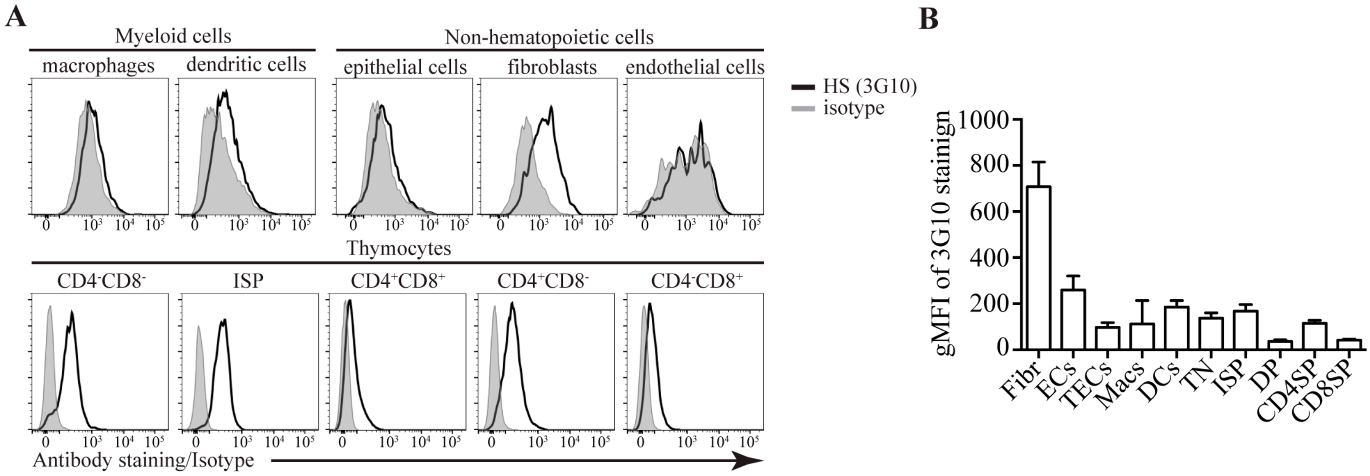

Figure S2. Expression of HS in the thymus determined with 3G10 antibody after HSase digestion. A. Flow cytometric detection of HS with 3G10 antibody in various cell types in the thymus (F4/80$^+$ macrophages, CD11c$^+$MHC2$^+$ dendritic cells, CD45$^-$EpCAM$^+$ epithelial cells, CD45$^-$EpCAM$^-$gp38$^+$ fibroblasts, CD45$^-$CD31$^+$ ECs, DN, DP, CD4SP, CD8SP and CD8$^+$CD4$^-$TCRβ$^-$ immature SP (ISP) thymocytes). The results are representative of 3 independent experiments.
B. Comparison of the amount of HS on the surfaces of the indicated non-hematopoietic cells revealed with 3G10 antibody. The results are mean±SEM of the geometric mean fluorescent intensity (gMFI) of HS staining from 3 independent experiments.

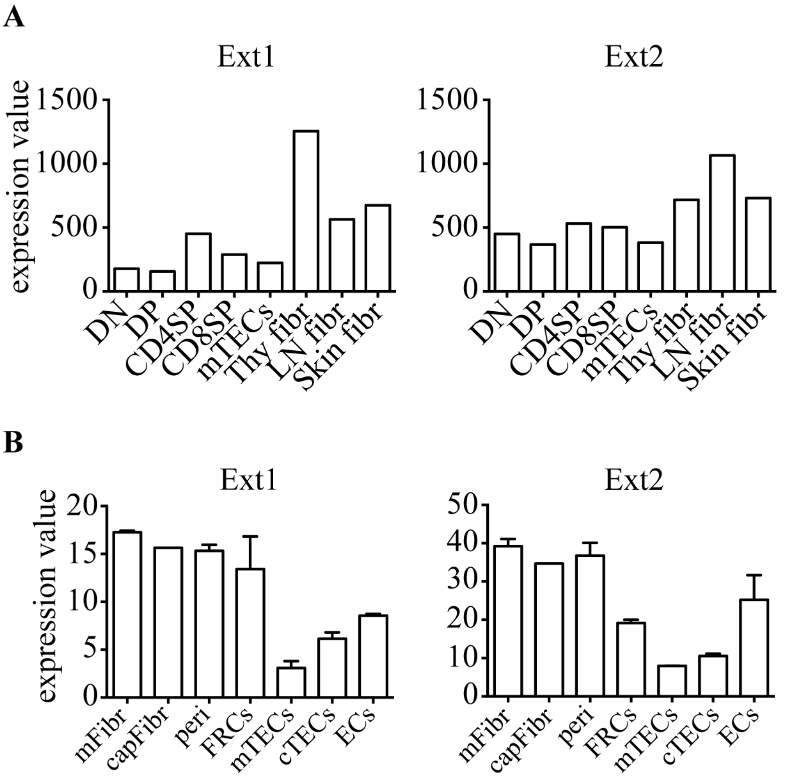

Figure S3. Expression of the glycosyltransferases Ext1 and Ext2 in cells in the thymus and fibroblasts in different organs. A. Mean expression data from IMMGEN Microarray database for Ext1 and Ext2 is plotted. The data for DN thymocytes is average of all DN populations (n=21). The data for DP is average of 6 samples. The data for CD4SP and CD8SP is the average of all subpopulations (n=9). The data for medullary TECs (mTECs) is average of 7 samples. Thymus fibroblasts (Thy fibr) are defined as CD45$^-$PDGFRα$^+$MTS15$^+$ cells (n=3). Skin fibroblasts (Skin fibr) are defined as CD45$^-$PDGFRα$^+$MTS15$^+$ cells (n=4). Lymph node fibroblasts (LN fibr) are defined as CD45$^-$gp38$^+$PDGFRα$^+$ fibroblastic reticular cells (n=2). **B.** Expression of Ext1 and Ext2 in thymic stromal cells and LM FRCs from RNA-Seq data from Nitta et al. Medullary fibroblasts (mFibr) are defined as CD45$^-$PDGFRαβ$^+$CD146$^-$gp38$^+$DPP4$^-$, capsular fibroblasts (capFibr) are CD45$^-$PDGFRαβ$^+$CD146$^-$gp38$^+$DPP4$^+$, pericytes (peri) are CD45-PDGFRαβ$^+$CD146$^+$gp38-, LN FRCs are CD45-gp38$^+$CD31-, mTECs are CD45$^-$EpCAM$^+$Ly51$^-$UEA1$^+$, cTECs are CD45$^-$EpCAM$^+$Ly51$^+$UEA1$^-$, and ECs are CD45$^-$CD31$^+$. The graphs show mean±SEM when more than 1 sample is available.

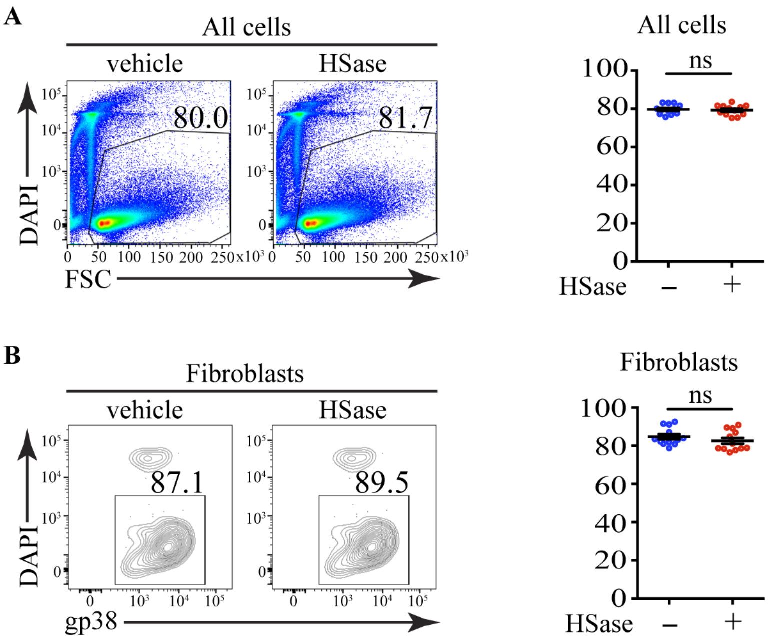

Figure S4: HSase treatment does not affect the viability of cells in the thymus, including fibroblasts. **A.** Flow cytometry staining for viability with DAPI of total cells in the thymus treated or not with HSase (left) and comparison of the viability between samples treated or not with HSase. **B.** DAPI staining of $CD45^{-}gp38^{+}$ thymic fibroblasts treated or not with HSase (left) and comparison of the viability of fibroblasts treated or not with HSase. The data are mean±SEM from 3 independent experiments with 4 samples in each. Each symbol is a single sample. Statistical significance was determined with unpaired t-test, ns – not significant.
